# GEO-CEOS stage 4 validation of the Satellite Image Automatic Mapper lightweight computer program for ESA Earth observation level 2 product generation - Part 1: Theory

**DOI:** 10.1080/23312041.2018.1467357

**Published:** 2018-06-10

**Authors:** Andrea Baraldi, Michael Laurence Humber, Dirk Tiede, Stefan Lang

**Affiliations:** 1 Department of Agricultural Sciences, University of Naples Federico II, Portici, Italy; 2 Department of Geographical Sciences, University of Maryland, College Park, MD, USA; 3 Department of Geoinformatics – Z_GIS, University of Salzburg, Salzburg, Austria; 4 Italian Space Agency (ASI), Rome, Italy; 5 University of Melbourne, Australia

**Keywords:** Artificial intelligence, binary relationship, Cartesian product, cognitive science, color naming, connected-component multilevel image labeling, deductive inference, Earth observation, land cover taxonomy, high-level (attentive) and low-level (pre-attentional) vision, hybrid inference, image classification, image segmentation, inductive inference, machine learning-from-data, outcome and process quality indicators, radiometric calibration, remote sensing, surface reflectance, thematic map comparison, top-of-atmosphere reflectance, two-way contingency table, unsupervised data discretization/vector quantization, validation

## Abstract

ESA defines as Earth Observation (EO) Level 2 information product a single-date multi-spectral (MS) image corrected for atmospheric, adjacency and topographic effects, stacked with its data-derived scene classification map (SCM), whose legend includes quality layers cloud and cloud-shadow. No ESA EO Level 2 product has ever been systematically generated at the ground segment. To fill the information gap from EO big data to ESA EO Level 2 product in compliance with the GEO-CEOS stage 4 validation (*Val*) guidelines, an off-the-shelf Satellite Image Automatic Mapper (SIAM) lightweight computer program was validated by independent means on an annual 30 m resolution Web-Enabled Landsat Data (WELD) image composite time-series of the conterminous U.S. (CONUS) for the years 2006–2009. The SIAM core is a prior knowledge-based decision tree for MS reflectance space hyperpolyhedralization into static color names. Typically, a vocabulary of MS color names in a MS data (hyper)cube and a dictionary of land cover (LC) class names in the scene-domain do not coincide and must be harmonized (reconciled). The present Part 1—Theory provides the multidisciplinary background of a priori color naming. The subsequent Part 2—Validation accomplishes a GEO-CEOS stage 4 *Val* of the test SIAM-WELD annual map time-series in comparison with a reference 30 m resolution 16-class USGS National Land Cover Data 2006 map, based on an original protocol for wall-to-wall thematic map quality assessment without sampling, where the test and reference maps feature the same spatial resolution and spatial extent, but whose legends differ and must be harmonized.

## PUBLIC INTEREST STATEMENT

Synonym of scene-from-image reconstruction and understanding, vision is an inherently ill-posed cognitive task; hence, it is difficult to solve and requires a priori knowledge in addition to sensory data to become better posed for numerical solution. In the inherently ill-posed cognitive domain of computer vision, this research was undertaken to validate by independent means a lightweight computer program for prior knowledge-based multi-spectral color naming, called Satellite Image Automatic Mapper (SIAM), eligible for automated near real-time transformation of large-scale Earth observation (EO) image datasets into European Space Agency (ESA) EO Level 2 information product, never accomplished to date at the ground segment. An original protocol for wall-to-wall thematic map quality assessment without sampling, where legends of the test and reference map pair differ and must be harmonized, was adopted. Conclusions are that SIAM is suitable for systematic ESA EO Level 2 product generation, regarded as necessary not sufficient pre-condition to transform EO big data into timely, comprehensive and operational EO value-adding information products and services.

## 1. Introduction

Jointly proposed by the intergovernmental Group on Earth Observations (GEO) and the Committee on Earth Observation Satellites (CEOS), the implementation plan for years 2005–2015 of the Global Earth Observation System of Systems (GEOSS) aimed at systematic transformation of multi-source Earth observation (EO) *big data* into timely, comprehensive and operational EO value-adding products and services (GEO, 2005), submitted to the GEO-CEOS Quality Assurance Framework for Earth Observation (QA4EO) calibration/validation (*Cal/Val*) requirements and suitable “to allow the access to the Right Information, in the Right Format, at the Right Time, to the Right People, to Make the Right Decisions” (Group on Earth Observation/Committee on Earth Observation Satellites (GEO-CEOS), 2010). In this definition of GEOSS, term *big data* identifies “a collection of data sets so large and complex that it becomes difficult to process using on-hand database management tools or traditional data processing applications. The big data challenges include capture, storage, search, sharing, transfer, analysis and visualization” (Wikipedia, 2018a), typically summarized as the five Vs of big data, specifically, volume, variety, velocity, veracity and value (IBM, 2016; Yang, Huang, Li, Liu, & Hu, 2017).

The GEOSS mission cannot be considered fulfilled by the remote sensing (RS) community to date. This is tantamount to saying the RS community is data-rich, but information-poor, a conjecture known as DRIP syndrome (Bernus & Noran, 2017). Before supporting this thesis with observations, the following definition is introduced. In this paper, an EO-IUS is defined in operating mode if and only if it scores “high” in every index of a minimally dependent and maximally informative (mDMI) set of EO outcome and process (OP) quantitative quality indicators (Q^2^Is), to be community-agreed upon to be used by members of the community, in agreement with the GEO-CEOS QA4EO *Cal/Val* guidelines (GEO-CEOS, 2010). A proposed instantiation of mDMI set of EO OP-Q^2^Is includes: (i) degree of automation, inversely related to human-machine interaction, (ii) effectiveness, e.g., thematic mapping accuracy, (iii) efficiency in computation time and in run-time memory occupation, e.g., inversely related to the number of system’s free-parameters to be user-defined based on heuristics, (iv) robustness (vice versa, sensitivity) to changes in input data, (v) robustness to changes in input parameters to be user-defined, (vi) scalability to changes in user requirements and in sensor specifications, (vii) timeliness from data acquisition to information product generation, (viii) costs in manpower and computer power, (ix) value, e.g., semantic value of output products, economic value of output services, etc. (Baraldi, 2017, 2009; Baraldi & Boschetti, 2012a, 2012b; Baraldi, Boschetti, & Humber, 2014; Baraldi et al., 2010a, 2010b; Baraldi, Gironda, & Simonetti, 2010c; Duke, 2016). According to the Pareto formal analysis of multi-objective optimization problems, optimization of an mDMI set of OP-Q^2^Is is an inherently-ill posed problem in the Hadamard sense (Hadamard, 1902), where many Pareto optimal solutions lying on the Pareto efficient frontier can be considered equally good (Boschetti, Flasse, & Brivio, 2004). Any EO-IUS solution lying on the Pareto efficient frontier can be considered in operating mode, therefore suitable to cope with the five Vs of spatio-temporal EO big data (Yang et al., 2017).

Stating that to date the RS community is affected by the DRIP syndrome is like saying that past and present EO image understanding systems (EO-IUSs) have been typically outpaced by the rate of data collection of EO imaging sensors, whose quality and quantity are ever-increasing at an apparently exponential rate related to the Moore law of productivity (National Aeronautics and SpaceAdministration (NASA), 2016a). In common practice, EO-IUSs are overwhelmed by sensory data they are unable to transform into EO value-adding information products and services, in compliance with the GEO-CEOS QA4EO *Cal/Val* guidelines (GEO-CEOS, 2010). If this conjecture holds true, then existing EO-IUSs cannot be considered in operating mode because unsuitable to cope with the five Vs of spatio-temporal EO big data (Yang et al., 2017). Several observations (true-facts) support this thesis. First, in 2012 the percentage of EO data ever downloaded from the European Space Agency (ESA) databases was estimated at about 10% or less (D’Elia 2012). This estimate is equal or superior (never inferior) to the percentage of ESA EO data ever used by the RS community. Since 2012, the same EO data exploitation indicator is expected to decrease, because any increase in productivity of existing EO-IUSs seems unable to match the exponential increase in the rate of collection of EO sensory data (NASA, 2016a). Second, EO-IUSs presented in the RS literature are typically assessed and compared based on the sole thematic mapping accuracy, which means their mDMI set of EO OP-Q^2^Is remains largely unknown to date (Baraldi & Boschetti, 2012a, 2012b). As a consequence, the RS literature is unable to contradict the thesis that no EO-IUS is available in operating mode. For example, when EO data-derived thematic maps were generated by EO-IUSs based on a supervised (labeled) data learning approach, at continental or global spatial extent and with estimated accuracy not inferior to a target mapping accuracy requirement, the most limiting factors turned out to be the cost, timeliness, quality and availability of adequate supervised training data samples collected from field sites, existing maps or geospatial data archives in tabular form (Gutman et al., 2004). Third, no ESA EO data-derived Level 2 information product has ever been systematically generated at the ground segment (DLR and VEGA 2011; ESA 2015). In the ESA definition, an EO Level 2 information product is a single-date multi-spectral (MS) image radiometrically calibrated into surface reflectance (SURF) values corrected for atmospheric, adjacency and topographic effects, stacked with its data-derived scene classification map (SCM), whose legend includes quality layers cloud and cloud-shadow, starting from an ESA EO Level 1 product geometrically corrected and radiometrically calibrated into top-of-atmosphere reflectance (TOARF) values (European Space Agency (ESA), 2015; Deutsches Zentrum für Luft- und Raumfahrt e.V. (DLR) and VEGA Technologies, 2011; CNES, 2015).

**Figure 1.**
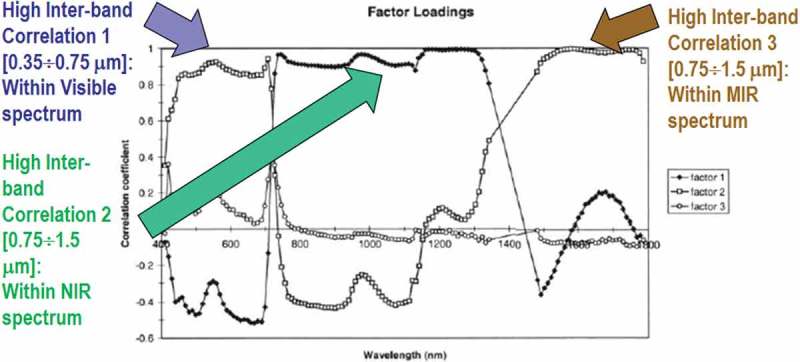
Courtesy of van der Meer and De John (2000). Pearson’s cross-correlation (CC) coefficients for the main factors resulting from a principal component analysis and factor rotation 1, 2 and 3 for an agricultural data set based on spectral bands of the AVIRIS hyper-spectral (HS) spectrometers. Flevoland test site, 5 July 1991. Inter-band CC values are “high” (>0.8) within the visible spectral range, the Near Infra-Red (NIR) wavelengths and the Medium IR (MIR) wavelengths. The general conclusion is that, irrespective of non-stationary local information, the global (image-wide) information content of a multi-channel image, either multi-spectral (MS) whose number *N* of spectral channels ∈ {2, 9}, super-spectral (SS) with *N* ∈ {10, 20}, or HS image with *N* > 20, can be preserved by selecting one visible band, one NIR band, one MIR band and one thermal IR (TIR) band, such as in the spectral resolution of the imaging sensor series National Oceanic and Atmospheric Administration (NOAA) Advanced Very High Resolution Radiometer (AVHRR), in operating mode from 1978 to date.

This last observation deserves further discussion. In the words of Marr, “vision goes symbolic almost immediately without loss of information” (Marr, 1982). In agreement with this Marr’s intuition, ESA defines as EO Level 2 information product an information primitive (unit of information) consisting of a stack of two coupled (inter-dependent) variables, one sub-symbolic/numeric and one symbolic, where symbolic variable means both categorical and semantics. Equivalent to two sides of the same coin, these two variables are very closely related to each other and cannot be separated, even though they seem different. The first side of the ESA EO Level 2 information primitive is a multivariate numeric variable of the highest radiometric quality, related to the concept of quantitative/unequivocal *information-as-thing* in the terminology of philosophical hermeneutics (Capurro & Hjørland, 2003), see Figure 1. The second side of the ESA EO Level 2 information unit is an EO data-derived SCM, equivalent to a categorical variable of semantic value, related to the concept of qualitative/equivocal *information-as-data-interpretation* in the terminology of philosophical hermeneutics (Capurro & Hjørland, 2003). In practice, ESA EO Level 2 product generation is a *chicken-and-egg* dilemma (Riano, Chuvieco, Salas, & Aguado, 2003), synonym of inherently ill-posed problem in the Hadamard sense (Hadamard, 1902). Therefore, it is very difficult to solve and requires a priori knowledge in addition to data to become better posed for numerical solution (Cherkassky & Mulier, 1998). On the one hand, no effective and efficient *Cal* of digital numbers (DNs) into SURF values corrected for atmospheric, topographic and adjacency effects is possible without an SCM, available *a priori* in addition to data to enforce a statistical stratification principle (Hunt & Tyrrell, 2012), synonym of layered (class-conditional) data analytics (Baraldi, 2017; Baraldi et al., 2010b; Baraldi & Humber, 2015; Baraldi, Humber, & Boschetti, 2013; Bishop & Colby, 2002; Bishop, Shroder, & Colby, 2003; DLR & VEGA, 2011; Dorigo, Richter, Baret, Bamler, & Wagner, 2009; Lück & van Niekerk, 2016; Riano et al., 2003; Richter & Schläpfer, 2012a, 2012b; Vermote & Saleous, 2007). On the other hand, no effective and efficient understanding (mapping) of a sub-symbolic EO image into a symbolic SCM is possible if DNs (pixels) are affected by low radiometric quality (GEO-CEOS, 2010). In an ESA EO Level 2 SCM product to be generated at the ground segment (midstream) as input to downstream applications, products and services (Mazzuccato & Robinson, 2017), the SCM legend is required to consist of a discrete, finite and hierarchical (multilevel) dictionary (Lipson, 2007; Mather, 1994; Swain & Davis, 1978) of general-purpose, user- and application-independent land cover (LC) classes, whose semantic value is “shallow” (not specialized) in hierarchy, but superior to zero semantics typical of numeric variables, in addition to quality layers cloud and cloud-shadow (ESA, 2015; DLR & VEGA, 2011; CNES, 2015). To our best knowledge, only one prototypical implementation of a sensor-specific ESA EO Level 2 product generator exists to date. Commissioned by ESA, the Sentinel 2 (atmospheric) Correction Prototype Processor (SEN2COR) is not run systematically at the ESA ground segment. Rather, it can be downloaded for free from the ESA web site to be run on user side (European Space Agency (ESA), 2015; Deutsches Zentrum für Luft- und Raumfahrt e.V. (DLR) and VEGA Technologies, 2011).

**Figure 2.**
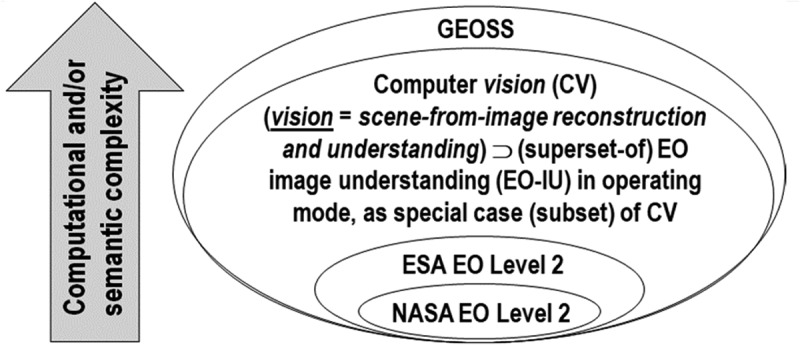
Graphical representation of a dependence relationship *part-of*, denoted with symbol “→” pointing from the supplier to the client in agreement with the standard Unified Modeling Language (UML) for graphical modeling of object-oriented software (Fowler, 2003), between computer vision (CV), whose special case is EO image understanding (EO-IU) in operating mode, where relationship *subset-of*, denoted with symbol “⊃” meaning specialization with inheritance from the superset to the subset, holds true, and a Global Earth Observation System of Systems (GEOSS) (GEO, 2005), such that “NASA EO Level 2 product → ESA EO Level 2 product ⊂ EO-IU in operating mode ⊂ CV → GEOSS”. Synonym of 4D spatio-temporal scene from (2D) image reconstruction and understanding, vision is acknowledged to be a cognitive problem very difficult to solve because: (i) non-polynomial (NP)-hard in computational complexity (Frintrop, 2011; Tsotsos, 1990), (ii) inherently ill-posed in the Hadamard sense, as it is affected by: (I) a 4D-to-2D data dimensionality reduction from the scene- to the image-domain, e.g., responsible of occlusion phenomena, and (II) a semantic information gap from ever-varying sub-symbolic sensory data (sensations) in the image-domain to stable symbolic percepts in the modeled world (mental world, world ontology, world model) (Fonseca et al., 2002; Laurini & Thompson, 1992; Matsuyama & Hwang, 1990; Sonka et al., 1994; Sowa, 2000). A NASA Earth observation (EO) Level 2 product, defined as “a data-derived geophysical variable at the same resolution and location as Level 1 source data” (NASA 2016b), is *part-of* the ESA EO Level 2 product, defined as follows (ESA, 2015; DLR & VEGA, 2011): (a) a single-date multi-spectral (MS) image whose digital numbers (DNs) are radiometrically calibrated into surface reflectance (SURF) values corrected for atmospheric, adjacency and topographic effects, stacked with (b) its data-derived general-purpose, user- and application-independent scene classification map (SCM), whose thematic map legend includes quality layers cloud and cloud-shadow (CNES, 2015). Working hypothesis “NASA EO Level 2 product → ESA EO Level 2 ⊂ EO-IU in operating mode → GEOSS” postulates that no GEOSS can exist if the necessary not sufficient pre-condition of systematic ESA EO Level 2 product generation is accomplished in advance as the mandatory first step in a hierarchical EO-IU workflow for scene-from-image reconstruction and understanding in operating mode.

Noteworthy, a National Aeronautics and Space Administration (NASA) EO Level 2 product is defined as “a data-derived geophysical variable at the same resolution and location as Level 1 source data” (NASA, 2016b). Hence, dependence relationship “NASA EO Level 2 product → ESA EO Level 2 product” holds, where symbol “→” denotes relationship *part-of* pointing from the supplier to the client, in agreement with the standard Unified Modeling Language (UML) for graphical modeling of object-oriented software (Fowler, 2003), see Figure 2. This dependence means that although space agencies and EO data distributors claim systematic NASA EO Level 2 product generation at the ground segment, this does not imply systematic ESA EO Level 2 product generation. Rather, the vice versa holds: if ESA EO Level 2 product generation is accomplished, then NASA EO Level 2 product generation is also fulfilled.

**Figure 3.**
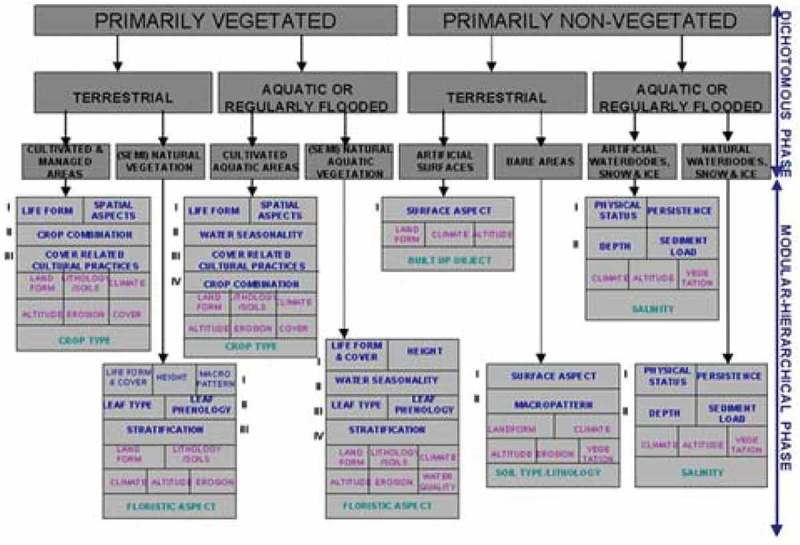
The fully nested 3-level 8-class FAO Land Cover Classification System (LCCS) Dichotomous Phase (DP) taxonomy consists of a sorted set of 3 dichotomous layers: (i) vegetation versus non-vegetation, (ii) terrestrial versus aquatic, and (iii) managed versus natural or semi-natural. They deliver as output the following 8-class LCCS-DP taxonomy. (A11) Cultivated and Managed Terrestrial (non-aquatic) Vegetated Areas. (A12) Natural and Semi-Natural Terrestrial Vegetation. (A23) Cultivated Aquatic or Regularly Flooded Vegetated Areas. (A24) Natural and Semi-Natural Aquatic or Regularly Flooded Vegetation. (B35) Artificial Surfaces and Associated Areas. (B36) Bare Areas. (B47) Artificial Waterbodies, Snow and Ice. (B48) Natural Waterbodies, Snow and Ice. The general-purpose user- and application-independent 3-level 8-class FAO LCCS-DP taxonomy is preliminary to a user- and application-specific FAO LCCS Modular Hierarchical Phase (MHP) taxonomy of one-class classifiers.

Different from the non-standard SEN2COR SCM legend instantiation (ESA, 2015; DLR & VEGA, 2011), one example of general-purpose, user- and application-independent ESA EO Level 2 SCM legend is the standard (community-agreed) 3-level 8-class dichotomous phase (DP) taxonomy of the Food and Agriculture Organization of the United Nations (FAO)—Land Cover Classification System (LCCS) (Di Gregorio & Jansen, 2000). The FAO LCCS-DP hierarchy is “fully nested”. It comprises three dichotomous LC class-specific information layers, equivalent to a world ontology or world model (Di Gregorio & Jansen, 2000; Matsuyama & Hwang, 1990): DP Level 1—Vegetation versus non-vegetation, DP Level 2—Terrestrial versus aquatic and DP Level 3—Managed versus natural or semi-natural. The 3-level 8-class FAO LCCS-DP taxonomy is shown in Figure 3. For the sake of generality, a 3-level 8-class FAO LCCS-DP legend is added with LC class “other”, synonym of “rest of the world” or “unknown”, which would include quality information layers cloud and cloud-shadow. In traditional EO image classification system design and implementation requirements (Swain & Davies, 1978), the presence of output class “unknown” is considered mandatory to cope with uncertainty in *information-as-data-interpretation* tasks. Hereafter, the standard 3-level 8-class FAO LCCS-DP legend added with the mandatory output class “other”, which includes quality layers cloud and cloud-shadow, is identified as “augmented” 9-class FAO LCCS-DP taxonomy. In the complete two-phase FAO LCCS hierarchy, a general-purpose 3-level 8-class FAO LCCS-DP legend is preliminary to a high-level application-dependent and user-specific FAO LCCS Modular Hierarchical Phase (MHP) taxonomy, consisting of a hierarchical (deep) battery of one-class classifiers (Di Gregorio & Jansen, 2000), see Figure 3. In recent years, the two-phase FAO LCCS taxonomy has become increasingly popular (Ahlqvist, 2008). One reason of this popularity is that the FAO LCCS hierarchy is “fully nested” while alternative LC class hierarchies, such as the Coordination of Information on the Environment (CORINE) Land Cover (CLC) taxonomy (Bossard, Feranec, & Otahel, 2000), the U.S. Geological Survey (USGS) Land Cover Land Use (LCLU) taxonomy by J. Anderson (Lillesand & Kiefer, 1979), the International Global Biosphere Programme (IGBP) DISCover Data Set Land Cover Classification System (Belward, 1996) and the EO Image Librarian LC class legend (Dumitru, Cui, Schwarz, & Datcu, 2015), start from a Level 1 taxonomy which is already multi-class. In a hierarchical EO-IUS architecture submitted to a *garbage in, garbage out* (GIGO) information principle, synonym of error propagation through an information processing chain, the fully-nested two-phase FAO LCCS hierarchy makes explicit the full dependence of high-level EO OP-Q^2^I estimates, featured by any high-level (deep) LCCS-MHP data processing module, on previous EO OP-Q^2^I values featured by lower-level LCCS modules, starting from the initial FAO LCCS-DP Level 1 vegetation/non-vegetation information layer whose relevance in thematic mapping accuracy (vice versa, in error propagation) becomes paramount for all subsequent LCCS layers. The GIGO commonsense principle applied to hierarchical semantic dependence is neither trivial nor obvious to underline (Marcus, 2018). On the one hand, it agrees with a minor portion of the RS literature where supervised data learning classification of EO image datasets at continental or global spatial extent into binary LC class vegetation/non-vegetation is considered very challenging (Gutman et al., 2004). On the other hand, it is at odd with the RS mainstream, where the semantic information gap from sub-symbolic EO data to multi-class LC taxonomies is typically filled in one step, implemented as a supervised data learning classifier (Bishop, 1995; Cherkassky & Mulier, 1998), e.g., a support vector machine, random forest or deep convolutional neural network (DCNN) (Cimpoi, Maji, Kokkinos, & Vedaldi, 2014), which is equivalent to an unstructured black box (Marcus, 2018), inherently semiautomatic and site specific (Liang, 2004) and whose opacity contradicts the well-known engineering principles of modularity, regularity and hierarchy typical of scalable systems (Lipson, 2007).

Starting from these premises our working hypothesis was that necessary not sufficient pre-condition for a yet-unfulfilled GEOSS development (GEO, 2005) is systematic generation at the ground segment of an ESA EO Level 2 product, never accomplished to date (ESA, 2015; DLR & VEGA, 2011), whose general-purpose SCM product is constrained as follows. First, the ESA EO Level 2 SCM legend agrees with the 3-level 9-class “augmented” FAO LCCS-DP taxonomy. Second, to comply with the GEO-CEOS QA4EO *Cal/Val* requirements, the SCM product must be submitted to a GEO-CEOS stage 4 *Val*, where an mDMI set of EO OP-Q^2^Is is evaluated by independent means at large spatial-extent and multiple time periods (GEO-CEOS, 2010). By definition, a GEO-CEOS stage 3 *Val* requires that “spatial and temporal consistency of the product with similar products are evaluated by independent means over multiple locations and time periods representing global conditions. In Stage 4 *Val*, results for Stage 3 are systematically updated when new product versions are released and as the time-series expands” (GEO-CEOS WGCV, 2015).

**Figure 4.**
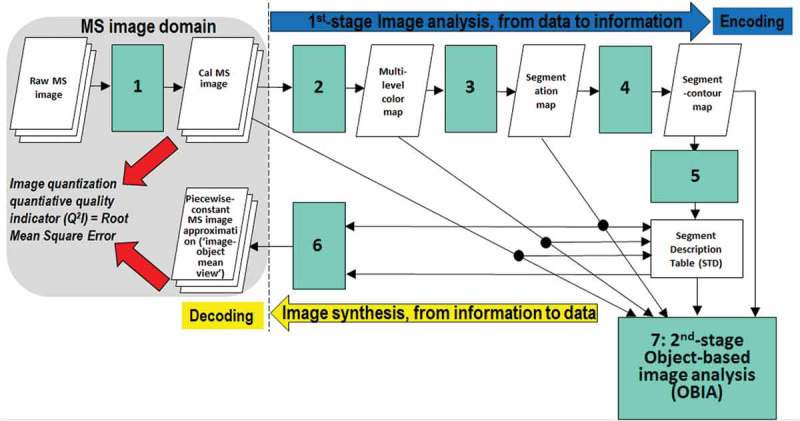
The SIAM lightweight computer program for prior knowledge-based MS reflectance space hyperpolyhedralization into color names, superpixel detection and vector quantization (VQ) quality assessment. It consists of six subsystems, identified as 1 to 6. Phase 1-of-2 = Encoding phase/Image analysis—Stage 1: MS data calibration into top-of-atmosphere reflectance (TOARF) or surface reflectance (SURF) values. Stage 2: Prior knowledge-based SIAM decision tree for MS reflectance space partitioning (quantization, hyperpolyhedralization). Stage 3: Well-posed (deterministic) two-pass connected-component detection in the multilevel color map-domain. Connected-components in the color map-domain are connected sets of pixels featuring the same color label. These connected-components are also called image-objects, segments or superpixels. Stage 4: Well-posed superpixel-contour extraction. Stage 5: Superpixel description table allocation and initialization. Phase 2-of-2 = Decoding phase/Image synthesis—Stage 6: Superpixelwise-constant input image approximation (“image-object mean view”) and per-pixel VQ error estimation. (Stage 7: in cascade to the SIAM superpixel detection, a high-level object-based image analysis (OBIA) approach can be adopted).

According to our working hypothesis, to contribute toward filling an analytic and pragmatic information gap from multi-source EO big data to ESA EO Level 2 information product as necessary not sufficient pre-condition to GEOSS development, the primary goal of this interdisciplinary study was to undertake an original (to the best of these authors’ knowledge, the first) outcome and process GEO-CEOS stage 4 *Val* of an off-the-shelf lightweight computer program, the Satellite Image Automatic Mapper™ (SIAM™), presented in recent years in the RS literature where enough information was provided for the implementation to be reproduced (Baraldi, 2017; Baraldi & Boschetti, 2012a, 2012b; Baraldi et al., 2010a, 2010b, 2010c; Baraldi & Humber, 2015; Baraldi et al., 2013; Baraldi, Puzzolo, Blonda, Bruzzone, & Tarantino, 2006, 2015; Baraldi, Tiede, Sudmanns, Belgiu, & Lang, 2016). Implemented in operating mode in the C/C++ programming language, an off-the-shelf SIAM software executable runs: (i) automatically, i.e., it requires no human-machine interaction, (iii) in near real-time because it is non-iterative, more specifically it is one-pass, with a single subsystem which is two-pass (refer to the text below), and its computational complexity increases linearly with image size, and (iii) in tile streaming mode, i.e., it requires a fixed run-time memory occupation. In addition to running on laptop and desktop computers, the SIAM lightweight computer program is eligible for use as mobile software application. By definition, a mobile software application is a lightweight computer program specifically designed to run on web services and/or mobile devices, such as tablet computers and smartphones, eventually provided with a mobile graphic user interface (GUI). An off-the-shelf SIAM software executable comprises six non-iterative subsystems for automated MS image analysis (decomposition) and synthesis (reconstruction) in linear time complexity. Its core is a one-pass prior knowledge-based decision tree (expert system) for MS reflectance space hyperpolyhedralization into static (non-adaptive-to-data) color names. Sketched in Figure 4, the SIAM software architecture is summarized as follows.

**Figure 5.**
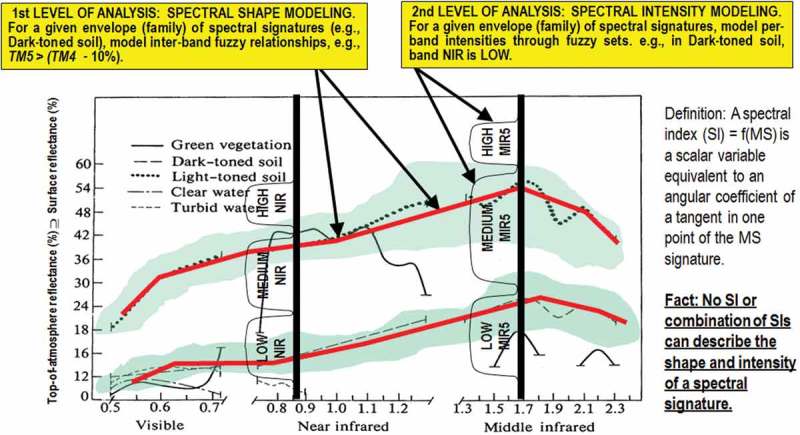
Examples of land cover (LC) class-specific families of spectral signatures in top-of-atmosphere reflectance (TOARF) values which include surface reflectance (SURF) values as a special case in clear sky and flat terrain conditions. A within-class family of spectral signatures (e.g., dark-toned soil) in TOARF values forms a buffer zone (hyperpolyhedron, envelope, manifold). The SIAM decision tree models each target family of spectral signatures in terms of multivariate shape and multivariate intensity information components as a viable alternative to multivariate analysis of spectral indexes. A typical spectral index is a scalar band ratio equivalent to an angular coefficient of a tangent in one point of the spectral signature. Infinite functions can feature the same tangent value in one point. In practice, no spectral index or combination of spectral indexes can reconstruct the multivariate shape and multivariate intensity information components of a spectral signature.

**Figure 6.**
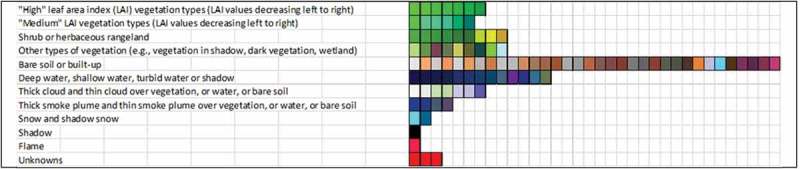
Prior knowledge-based color map legend adopted by the Landsat-like SIAM (L-SIAM™, release 88 version 7) implementation. For the sake of representation compactness, pseudo-colors of the 96 spectral categories are gathered along the same raw if they share the same parent spectral category in the decision tree, e.g., “strong” vegetation, equivalent to a spectral end-member. The pseudo-color of a spectral category is chosen as to mimic natural colors of pixels belonging to that spectral category. These 96 color names at fine color granularity are aggregated into 48 and 18 color names at intermediate and coarse color granularity respectively, according to parent–child relationships defined *a priori*, also refer to Table 1.

**Figure 7.**
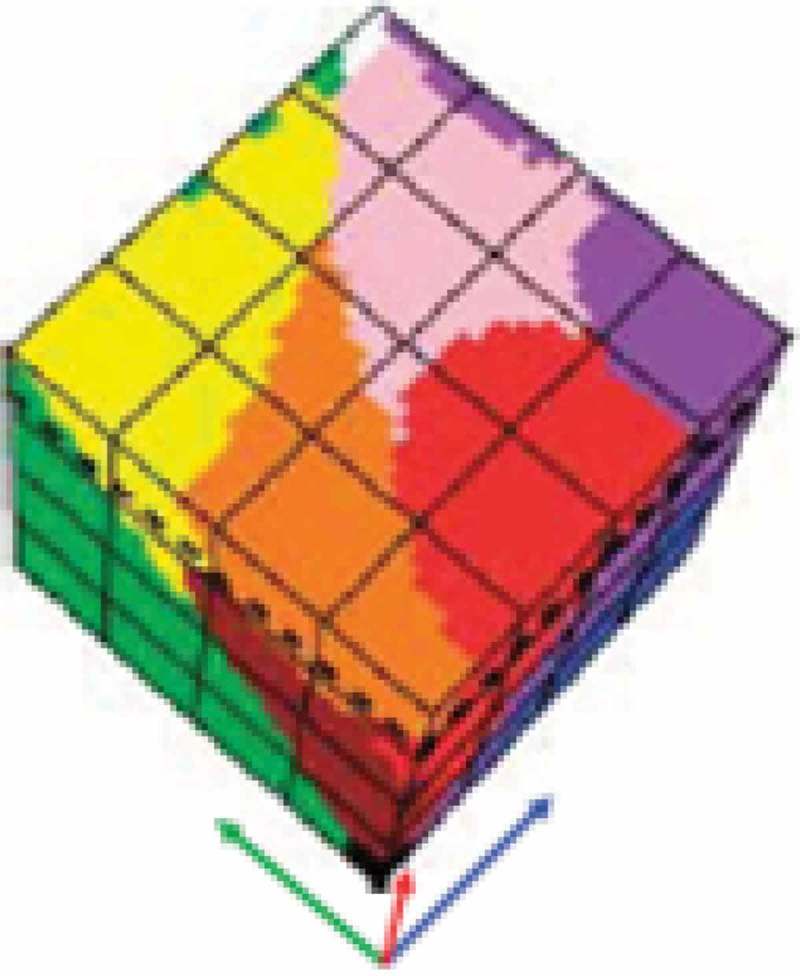
Courtesy of Griffin (2006). Monitor-typical RGB cube partitioned into perceptual polyhedra corresponding to a discrete and finite dictionary of basic color (BC) names, to be community-agreed upon in advance to be employed by members of the community. The mutually exclusive and totally exhaustive polyhedra are neither necessarily convex nor connected. In practice BC names belonging to a finite and discrete color vocabulary are equivalent to Vector Quantization (VQ) levels belonging to a VQ codebook (Cherkassky & Mulier, 1998).

**Figure 8.**
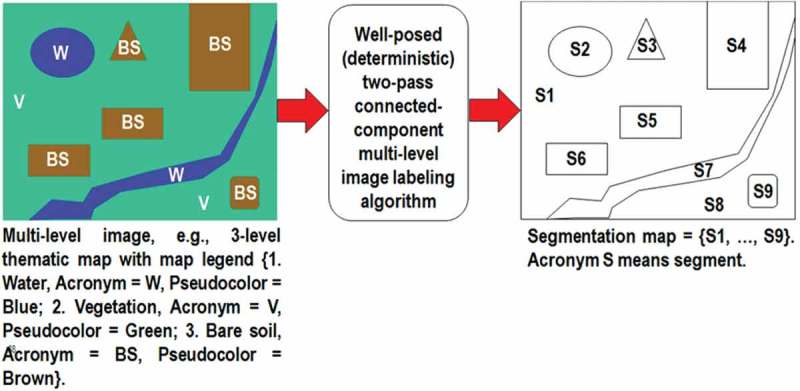
One segmentation map is deterministically generated from one multilevel image, such as a thematic map, but the vice versa does not hold, i.e., many multilevel images can generate the same segmentation map. In this example, nine image-objects/segments S1–S9 can be detected in the 3-level thematic map shown at left. Each segment consists of a connected set of pixels sharing the same multilevel map label. Each stratum/layer/level consists of one or more segments, e.g., stratum Vegetation (V) consists of two disjoint segments, S1 and S8. In any multilevel (categorical, nominal, qualitative) image domain, three labeled spatial primitives (spatial units) coexist and are provided with parent–child relationships: pixel with a level-label and a pixel identifier (ID, e.g., the row-column coordinate pair), segment (polygon) with a level-label and a segment ID, and stratum (multi-part polygon) with a level-label equivalent to a stratum ID. This overcomes the ill-fated dichotomy between traditional unlabeled sub-symbolic pixels versus labeled sub-symbolic segments in the numeric (quantitative) image domain traditionally coped with by the object-based image analysis (OBIA) paradigm (Blaschke et al., 2014).

**Figure 9.**
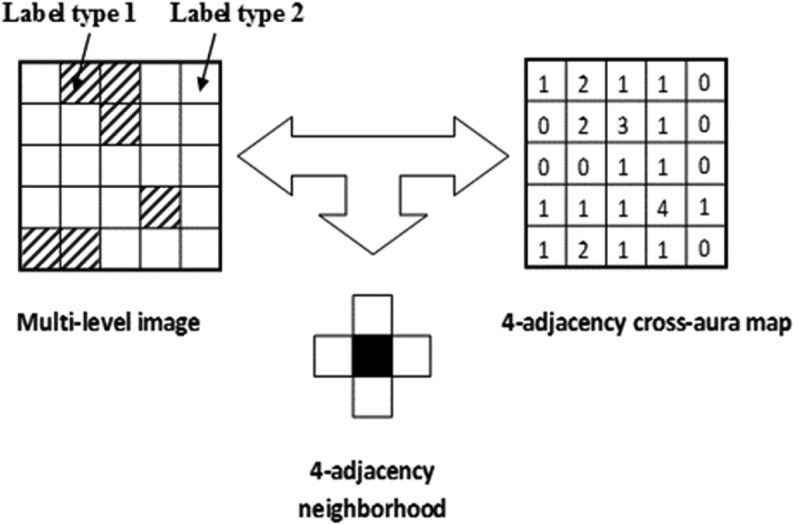
Example of a 4-adjacency cross-aura map, shown at right, generated in linear time from a two-level image shown at left.

1. MS data radiometric calibration, in agreement with the GEO-CEOS QA4EO *Cal* requirements (GEO-CEOS, 2010). The SIAM expert system instantiates a physical data model; hence, it requires as input sensory data provided with a physical meaning. Specifically, DNs must be radiometrically *Cal* into a physical unit of radiometric measure to be community-agreed upon, such as TOARF values, SURF values or Kelvin degrees for thermal channels. Relationship TOARF ⊇ SURF holds because SURF is a special case of TOARF in clear sky and flat terrain conditions (Chavez, 1988), i.e., TOARF ≈ SURF + atmospheric noise + topographic effects + surface adjacency effects. In a spectral decision tree for MS color space hyperpolyhedralization (partitioning), this relationship means that MS hyperpolyhedra (envelopes, manifolds) in “noisy” TOARF values include “noiseless” hyperpolyhedra in SURF values as special case of the former according to relationship *subset-of*, while the vice versa does not hold, see Figure 5.
2. One-pass prior knowledge-based SIAM decision tree for MS reflectance space hyperpolyhedralization into three static codebooks (vocabularies) of sub-symbolic/semi-symbolic color names as codewords, see Figure 5. Provided with inter-level parent–child relationships, the SIAM’s three-level vocabulary of static color names features a ColorVocabularyCardinality value which decreases from fine to intermediate to coarse, refer to Table 1 and Figure 6. MS reflectance space hyperpolyhedra for color naming are difficult to think of and impossible to visualize when the MS data space dimensionality is superior to three. This is not the case of basic color (BC) names adopted in human languages (Berlin & Kay, 1969), whose mutually exclusive and totally exhaustive perceptual polyhedra, neither necessarily convex nor connected, are intuitive to think of and easy to visualize in a 3D monitor-typical red-green-blue (RGB) data cube, see Figure7 (Benavente, Vanrell, & Baldrich, 2008; Griffin, 2006). When each pixel of an MS image is mapped onto a color space partitioned into a set of mutually exclusive and totally exhaustive hyperpolyhedra equivalent to a vocabulary of BC names, then a 2D multilevel color map (2D gridded dataset of a multilevel variable) is generated automatically (without human-machine interaction) in near real-time (with computational complexity increasing linearly with image size), where the number *k* of 2D map levels (color strata, color names) belongs to range {1, ColorVocabularyCardinality}. Popular synonyms of measurement space hyperpolyhedralization (discretization, partition) are vector quantization (VQ) in inductive machine learning-from-data (Cherkassky & Mulier, 1998; Elkan, 2003; Fritzke, 1997a, 1997b; Lee, Baek, & Sung, 1997; Linde, Buzo, & Gray, 1980; Lloyd, 1982; Patanè and Russo, 2001, 2002), and deductive fuzzification of a numeric variable into fuzzy sets in fuzzy logic (Zadeh, 1965). Typical inductive learning-from-data VQ algorithms aim at minimizing a known VQ error function, e.g., a root mean square vector quantization error (RMSE), given a number of *k* discretization levels selected by a user based on *a priori* knowledge and/or heuristic criteria. One of the most widely used VQ heuristics in RS and computer vision (CV) applications is the *k*-means VQ algorithm (Elkan, 2003; Lee et al., 1997; Linde et al., 1980; Lloyd, 1982), capable of convex Voronoi tessellation of a multi-variate data space (Cherkassky & Mulier, 1998; Fritzke, 1997a). For example, in a bag-of-words model applied to CV tasks, a numeric color space is typically discretized into a categorical color variable (codebook of codewords) by an inductive VQ algorithm, such as *k*-means; next, the categorical color variable is simplified by a 1st-order histogram representation, which disregards word grammar, semantics and even word-order, but keeps multiplicity; finally, the frequency of each color codeword is used as a feature for training a supervised data learning classifier (Cimpoi et al., 2014). Unlike the *k*-means VQ algorithm where the system’s free-parameter *k* is user-defined based on heuristics and the VQ error is estimated from the unlabeled dataset at hand, a user can fix the target VQ error value, so that it is the free-parameter *k* to be dynamically learned from the finite unlabeled dataset at hand by an inductive VQ algorithm (Patané & Russo, 2001, 2002), such as ISODATA (Memarsadeghi, Mount, Netanyahu, & Le Moigne, 2007). It means there is no universal number *k* of static hyperpolyhedra in a vector data space suitable for satisfying any VQ problem of interest if no target VQ error is specified in advance. As a viable strategy to cope with the inherent ill-posedness of inductive VQ problems (Cherkassky & Mulier, 1998), the SIAM expert system provides its three pre-defined VQ levels with a per-pixel RMSE estimation required for VQ quality assurance, in compliance with the GEO-CEOS QA4EO *Val* guidelines, refer to point (6) below.
3. Well-posed (deterministic) two-pass detection of connected-components in the multilevel color map-domain (Dillencourt, Samet, & Tamminen, 1992; Sonka, Hlavac, & Boyle, 1994), where the number *k* of map levels belongs to range {1, ColorVocabularyCardinality}, see Figure 8. These discrete and finite connected-components consist of connected sets of pixels featuring the same color label. Each connected-component is either (0D) pixel, (1D) line or (2D) polygon in the Open Geospatial Consortium (OGC) nomenclature (OGC 2015). They are typically known as *superpixels* in the CV literature (Achanta et al., 2011), homogeneous *segments* or *image-objects* in the object-based image analysis (OBIA) literature (Blaschke et al., 2014; Matsuyama & Hwang, 1990; Nagao & Matsuyama, 1980; Shackelford & Davis, 2003a, 2003b), and texture elements, i.e., *texels*, in human vision (Julesz, 1986; Julesz, Gilbert, Shepp, & Frisch, 1973). Whereas the physical model-based SIAM expert system requires no human-machine interaction to detect top-down superpixels whose shape and size can be any, superpixels detected bottom-up in statistical model-based CV algorithms typically require a pair of statistical model’s free-parameters to be user-defined based on heuristics, such as a first heuristic-based geometric threshold equal to the superpixel maximum area and a second heuristic-based geometric threshold forcing a superpixel to stay compact in shape (Achanta et al., 2011). In a multilevel image domain where *k* is the number of levels (image-wide strata), individual (0D) pixels with label 1 to *k*, superpixels as connected sets of pixels featuring the same label 1 to *k*, and strata (layers), equal to discrete and finite collections of superpixels, mutually disjoint, but belonging to the same level 1 to *k*, co-exist as non-alternative labeled spatial units provided with a parent–child relationship, where each superpixel is a 2-tuple (superpixel ID, level 1-of-*k*) and each pixel is a 2-tuple (raw-column coordinate pair, superpixel ID), see Figure 8.
4. Well-posed 4- or 8-adjacency cross-aura representation in linear time of superpixel-contours, see Figure 9. These cross-aura contour values allow estimation of a scale-invariant planar shape index of compactness (Baraldi, 2017; Baraldi & Soares, 2017; Soares, Baraldi, & Jacobs, 2014), eligible for use by a high-level OBIA approach (Blaschke et al., 2014), see Figure 4.
5. Superpixel/segment description table (Matsuyama & Hwang, 1990; Nagao & Matsuyama, 1980), to describe superpixels in a 1D tabular form (list) in combination with their 2D raster representation in the image-domain, referred to as “literal bit map” by Marr (1982), to take advantage of each data structure and overcome their shortcomings. Computationally, local spatial searches are more efficient in the 2D raster image-domain than in the 1D list representation, because “most of the spatial relationships that must be examined in early vision (encompassing the raw and full primal sketch for token detection and texture segmentation, respectively) are rather local” (Marr, 1982). Vice versa, if we had to examine global or “scattered, pepper-and-salt-like (spatial) configurations, then a (2D) bit map would probably be no more efficient than a (1D) list” (Marr, 1982).
6. Superpixelwise-constant input image approximation (reconstruction), also known as “image-object mean view” in commercial OBIA applications (Trimble, 2015), followed by a per-pixel RMSE estimation between the original MS image and the reconstructed piecewise-constant MS image. This VQ error estimation strategy enforces a product quality assurance policy considered mandatory by the GEO-CEOS QA4EO *Val* guidelines. For example, VQ quality assurance supported by SIAM allows a user to adopt quantitative (objective) criteria in the selection of pre-defined VQ levels, equivalent to color names, to fit user- and application-specific VQ error requirement specifications.

**Table 1.** The SIAM computer program is an EO system of systems scalable to any past, existing or future MS imaging sensor provided with radiometric calibration metadata parameters

SIAM, r88v7	Input bands	Prior knowledge-based color map legends: Number of output spectral categories = Vocabulary of multi-spectral (MS) color names			
		Fine discretization levels	Intermediate discretization levels	Coarse discretization levels	Inter-sensor discretization levels (*)
**L-SIAM**	**7—B, G, R, NIR, MIR1, MIR2, TIR**	**96**	**48**	**18**	**33** (*): employed for inter-sensor post-classification change/no-change detection
**S-SIAM**	**4—G, R, NIR, MIR1**	**68**	**40**	**15**	
**AV-SIAM**	**4—R, NIR, MIR1, TIR**	**83**	**43**	**17**	
**Q-SIAM**	**4—B, G, R, NIR**	**61**	**28**	**12**	

It encompasses the following subsystems. (i) 7-band Landsat-like SIAM™ (L-SIAM™), with input channels Blue (B), Green (G), Red (R), Near Infra-Red (NIR), Medium IR1 (MIR1), Medium IR2 (MIR2), and Thermal IR (TIR). (ii) 4-band (channels G, R, NIR, MIR1) SPOT-like SIAM™ (S-SIAM™). (iii) 4-band (channels R, NIR, MIR1, and TIR) Advanced Very High Resolution Radiometer (AVHRR)-like SIAM™ (AV-SIAM™). (iv) 4-band (channels B, G, R, and NIR) QuickBird-like SIAM™ (Q-SIAM™)

**Figure 10.**
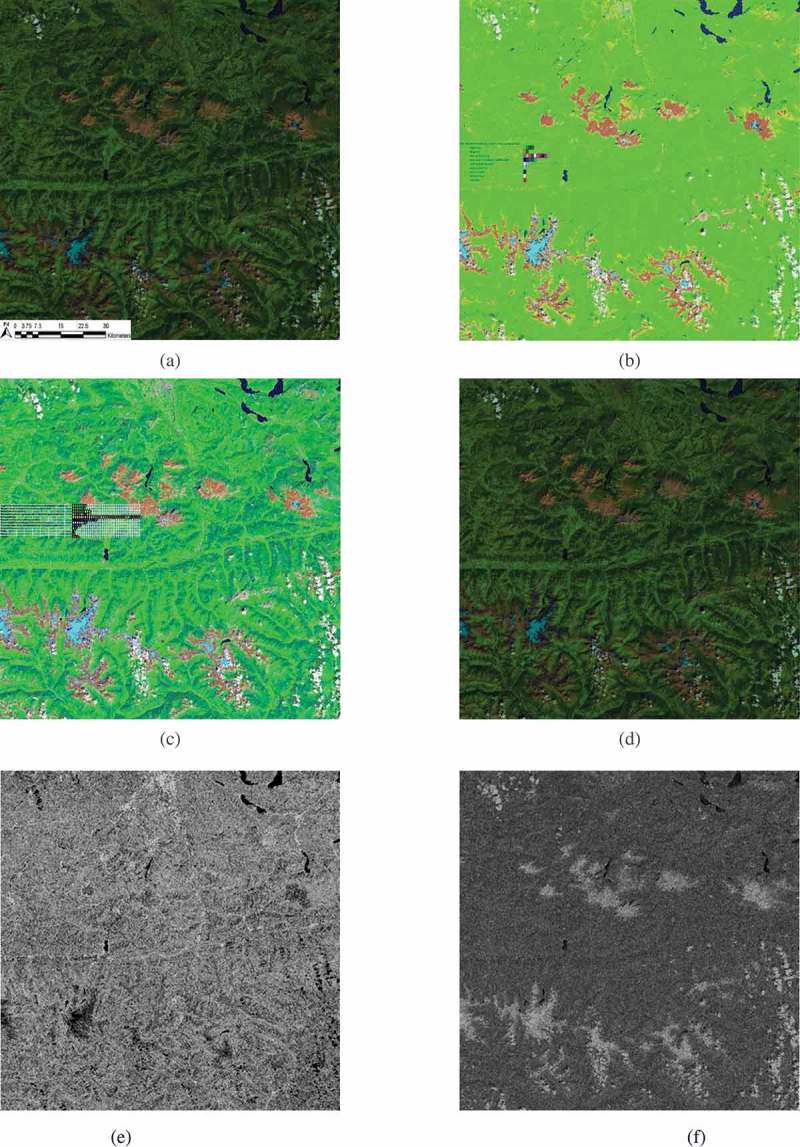
(a) Sentinel-2A (S2A) MSI Level-1C image radiometrically calibrated (*Cal*) into top-of-atmosphere reflectance (TOARF) values by the ESA data provider, depicting an Earth surface located south of the city of Salzburg, Austria. The city area is visible around the middle of the image upper boundary (Lat-long coordinates: 47°48ʹ25.0ʺN 13°02ʹ43.6ʺE). Acquired on 2015–09-11. Spatial resolution: 10 m. Image size: 110 × 110 km. TOARF values in range [0, 1] are byte-coded in range {0, 255}. The MS image is shown in RGB false colors, where monitor channel R = Medium InfraRed (MIR) = S2 Band 11, channel G = Near IR (NIR) = S2 Band 8, channel B = (visible) Blue = S2 Band 2. No histogram stretching is applied for visualization purposes. (b) L-SIAM color map at coarse color granularity, consisting of 18 spectral categories depicted in pseudo colors shown in the map legend. Coarse-granularity color categories are generated by merging color hyperpolyhedra at fine color granularity, according to pre-defined parent–child relationships, refer to Table 1. (c) L-SIAM color map at fine color granularity, consisting of 96 spectral categories depicted in pseudo colors shown in the map legend, refer to Table 1. (d) Superpixelwise-constant approximation of the input image (“image-object mean view”) generated from the L-SIAM’s 96 color map at fine granularity. Depicted in false colors: R = MIR = S2 Band 11, G = NIR = S2 Band 8, B = (visible) Blue = S2 Band 2. Spatial resolution: 10 m. No histogram stretching is applied for visualization purposes. (e) 8-adjacency cross-aura contour map in range {0, 8} automatically generated from the L-SIAM’s 96 color map at fine granularity. It shows contours of connected sets of pixels featuring the same color label. These connected-components are also called image-objects, segments or superpixels. (f) Per-pixel scalar difference between the input MS image shown in (a) and the superpixelwise-constant MS image reconstruction shown in (d). This scalar difference is computed as the per-pixel Root Mean Square Error (RMSE) in range {0, 255}, which is the same domain of change of input byte-coded pixel values. The RMSE is a well-known vector quantization (VQ) error estimate to be minimized. Image-wide RMSE statistics: Min = 0, Max = 130, Mean = 2.60, Stdev = 3.45. Histogram stretching is applied for visualization purposes. The highest RMSE values are located in pixels belonging to segments labeled as snow and cloud, which tend to be larger in size and whose class-specific within-segment variance tends to be “high”.

An example of the SIAM output products automatically generated in linear time from a 13-band 10 m-resolution Sentinel-2A image radiometrically calibrated into TOARF values is shown in Figure 10.

**Figure 11.**
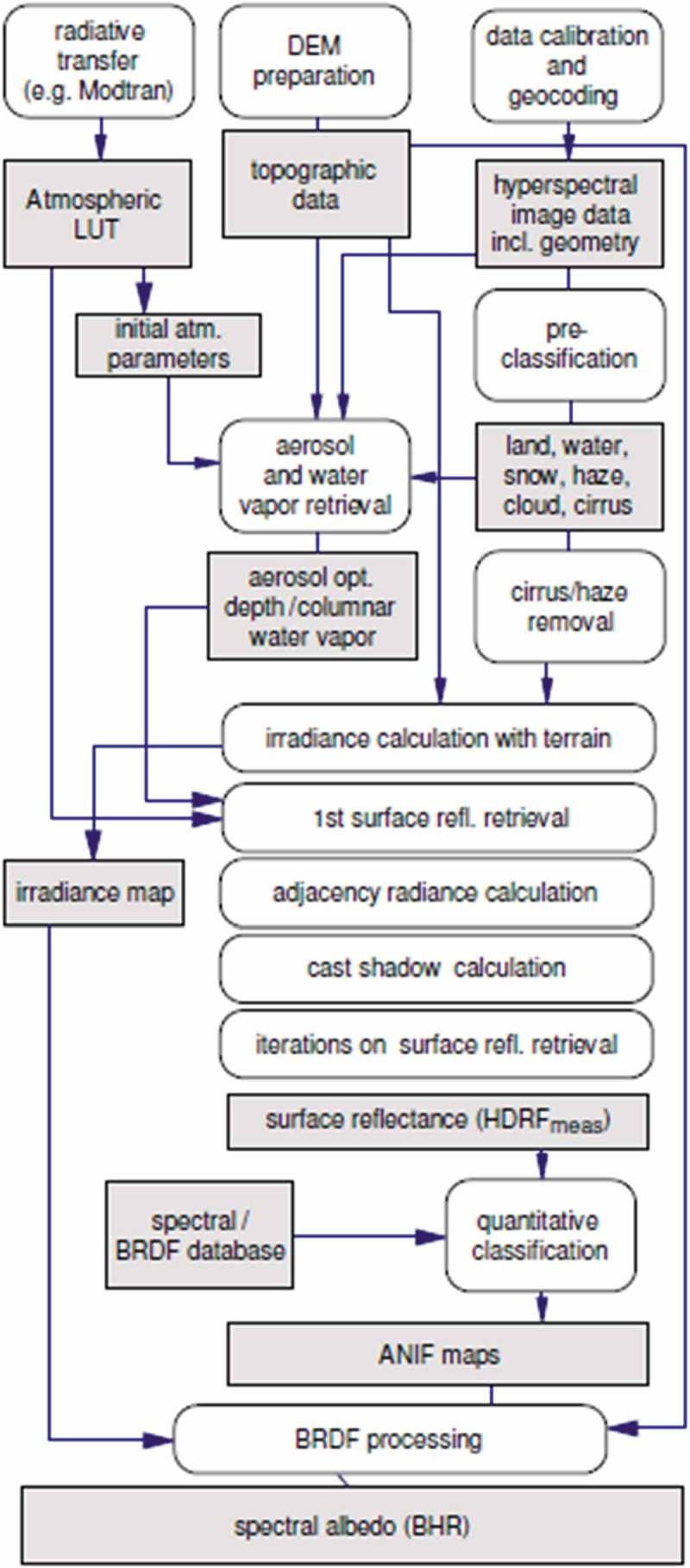
Same as in Schläpfer et al. (2009), courtesy of Daniel Schläpfer, ReSe Applications Schläpfer. A complete (“augmented”) hybrid inference workflow for MS image correction from atmospheric, adjacency and topographic effects. It combines a standard Atmospheric/Topographic Correction for Satellite Imagery (ATCOR) commercial software workflow (Richter & Schläpfer, 2012a, 2012b), with a bidirectional reflectance distribution function (BRDF) effect correction. Processing blocks are represented as circles and output products as rectangles. This hybrid (combined deductive and inductive) workflow alternates deductive/prior knowledge-based with inductive/learning-from-data inference units, starting from initial conditions provided by a first-stage deductive Spectral Classification of surface reflectance signatures (SPECL) decision tree for color naming (pre-classification), implemented within the ATCOR commercial software toolbox (Richter & Schläpfer, 2012a, 2012b). Categorical variables generated by the pre-classification and classification blocks are employed to stratify (mask) unconditional numeric variable distributions, in line with the statistic stratification principle (Hunt & Tyrrell, 2012). Through statistic stratification (class-conditional data analytics), inherently ill-posed inductive learning-from-data algorithms are provided with prior knowledge required in addition to data to become better posed for numerical solution, in agreement with the machine learning-from-data literature (Cherkassky & Mulier, 1998).

**Figure 12.**
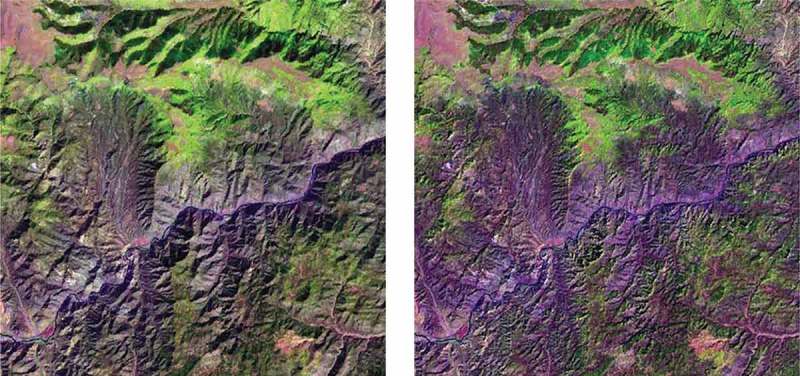
Left: Zoomed area of a Landsat 7 ETM+ image of Colorado, USA (path: 128, row: 021, acquisition date: 2000–08-09), depicted in false colors (R: band ETM5, G: band ETM4, B: band ETM1), 30 m resolution, radiometrically calibrated into TOARF values. Right: Output product automatically generated without human-machine interaction by the stratified topographic correction (STRATCOR) algorithm proposed in Baraldi et al. (2010c), whose input datasets are one Landsat image, its data-derived L-SIAM color map at coarse color granularity, consisting of 18 spectral categories for stratification purposes (see Table 1), and a standard 30 m resolution Shuttle Radar Topography Mission (SRTM) digital elevation model (DEM).

The potential impact on the RS community of a GEO-CEOS stage 4 *Val* of an off-the-shelf SIAM lightweight computer program for automated near real-time prior knowledge-based MS reflectance space hyperpolyhedralization, superpixel detection and per-pixel VQ quality assessment is expected to be relevant, with special emphasis on existing or future *hybrid* (combined deductive and inductive) EO-IUSs. In the RS discipline, there is a long history of prior knowledge-based MS reflectance space partitioners for static color naming, alternative to SIAM’s, developed but never validated by space agencies, public organizations and private companies for use in hybrid EO-IUSs in operating mode, see Figure 11. Examples of hybrid EO image pre-processing applications in the quantitative/sub-symbolic domain of *information-as-thing*, where a numeric input variable is statistically class-conditioned (masked) by a static color naming first stage to generate as output another numeric variable considered more informative than the input one,  are large-scale MS image compositing (Ackerman et al. 1998; Lück & van Niekerk, 2016; Luo, Trishchenko, & Khlopenkov, 2008), MS image atmospheric correction and topographic correction (Baraldi, 2017; Baraldi et al., 2010b; Baraldi & Humber, 2015; Baraldi et al., 2013; Bishop & Colby, 2002; Bishop et al., 2003; DLR & VEGA, 2011; Dorigo et al., 2009; Lück & van Niekerk, 2016; Riano et al., 2003; Richter & Schläpfer, 2012a, 2012b; Vermote & Saleous, 2007), see Figure 12, MS image adjacency effect correction (DLR & VEGA, 2011) and radiometric quality assessment of pan-sharpened MS imagery (Baraldi, 2017; Despini, Teggi, & Baraldi, 2014). Examples of hybrid EO image classification applications in the qualitative/equivocal/categorical domain of *information-as-data-interpretation* and statistically class-conditioned by a static color naming first stage are cloud and cloud-shadow quality layer detection (Baraldi, 2015, 2017; Baraldil., DLR & VEGA, 2011; Lück & van Niekerk, 2016), single-date LC classification (DLR & VEGA, 2011; GeoTerraImage, 2015; Lück & van Niekerk, 2016; Muirhead & Malkawi, 1989; Simonetti et al. 2015a), multi-temporal post-classification LC change (LCC)/no-change detection (Baraldi, 2017; Baraldi et al., 2016; Simonetti et al., 2015a; Tiede, Baraldi, Sudmanns, Belgiu, & Lang, 2016), multi-temporal vegetation gradient detection and quantization into fuzzy sets (Arvor, Madiela, & Corpetti, 2016), multi-temporal burned area detection (Boschetti, Roy, Justice, & Humber, 2015), and prior knowledge-based LC mask refinement (cleaning) of supervised data samples employed as input to supervised data learning EO-IUSs (Baraldi et al., 2010a, 2010b). Due to their large application domain, ranging from low- (pre-attentional) to high-level (attentional) vision tasks, existing hybrid EO-IUSs in operating mode, whose statistical data models are class-conditioned by static color naming, become natural candidates for the research and development (R&D) of an EO-IUS in operating mode, capable of systematic transformation of multi-source single-date MS imagery into ESA EO Level 2 product at the ground segment.

**Figure 13.**
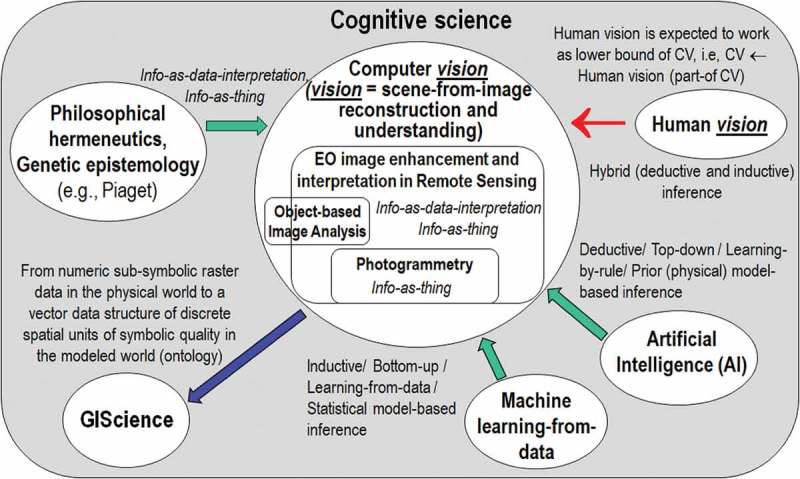
Like engineering, remote sensing (RS) is a metascience, whose goal is to transform knowledge of the world, provided by other scientific disciplines, into useful user- and context-dependent solutions in the world. Cognitive science is the interdisciplinary scientific study of the mind and its processes. It examines what cognition (learning) is, what it does and how it works. It especially focuses on how information/knowledge is represented, acquired, processed and transferred within nervous systems (distributed processing systems in humans, such as the human brain, or other animals) and machines (e.g., computers). Neurophysiology studies nervous systems, including the brain. Human vision is expected to work as lower bound of CV, i.e., human vision → (*part-of*) CV, such that inherently ill-posed CV is required to comply with human visual perception phenomena to become better conditioned for numerical solution.

The terminology adopted in the rest of this paper is mainly driven from the multidisciplinary domain of cognitive science, see Figure 13. Popular synonyms of deductive inference are top-down, prior knowledge-based, learning-from-rule and physical model-based inference. Synonyms of inductive inference are bottom-up, learning-from-data, learning-from-examples and statistical model-based inference (Baraldi, 2017; Baraldi & Boschetti, 2012a, 2012b; Liang, 2004). Hybrid inference systems combine statistical and physical data models to take advantage of the unique features of each and overcome their shortcomings (Baraldi, 2017; Baraldi & Boschetti, 2012a, 2012b; Cherkassky & Mulier, 1998; Liang, 2004). For example, in biological cognitive systems “there is never an absolute beginning” (Piaget, 1970), where an *a priori* genotype provides initial conditions to an inductive learning-from-examples phenotype (Parisi, 1991). Hence, any biological cognitive system is a hybrid inference system where inductive/phenotypic learning-from-examples mechanisms explore the neighborhood of deductive/genotypic initial conditions in a solution space (Parisi, 1991). In line with biological cognitive systems, an artificial hybrid inference system can alternate deductive and inductive inference algorithms, starting from a deductive inference first stage for initialization purposes, see Figure 11. It means that no deductive inference subsystem, such as SIAM, should be considered stand-alone, but eligible for use in a hybrid inference system architecture to initialize (pre-condition, stratify) inductive learning-from-data algorithms, which are inherently ill-posed, difficult to solve and require *a priori* knowledge in addition to data to become better posed for numerical solution, as clearly acknowledged by the machine learning-from-data literature (Bishop, 1995; Cherkassky & Mulier, 1998).

To comply with the GEO-CEOS stage 4 *Cal*/*Val* requirements, the selected ready-for-use SIAM software executable had to be validated by independent means on a radiometrically calibrated EO image time-series at large spatial extent. This input data set was identified in the open-access USGS 30 m resolution Web Enabled Landsat Data (WELD) annual composites of the conterminous U.S. (CONUS) for the years 2006–2009, radiometrically calibrated into TOARF values (Homer, Huang, Yang, Wylie, & Coan, 2004; Roy et al., 2010; WELD 2015). The 30 m resolution 16-class U.S. National Land Cover Data (NLCD) 2006 map, delivered in 2011 by the USGS Earth Resources Observation Systems (EROS) Data Center (EDC) (EPA 2007; Vogelmann et al., 2001; Vogelmann, Sohl, Campbell, & Shaw, 1998; Wickham, Stehman, Fry, Smith, & Homer, 2010; Wickham et al., 2013; Xian & Homer, 2010), was selected as reference thematic map at continental spatial extent. The USGS 16-class NLCD 2006 map legend is summarized in Table 2. To account for typical non-stationary geospatial statistics, the USGS NLCD 2006 thematic map was partitioned into 86 Level III ecoregions of North America collected from the Environmental Protection Agency (EPA) (EPA 2013; Griffith & Omernik, 2009).

**Table 2.** Definition of the USGS NLCD 2001/2006/2011 classification taxonomy, Level II. ^2^ Alaska only

USGS NLCD 2001/2006/2011 Classification Scheme (Legend), Level II				FAO LCCS-DP, level 1: A = Veg, B = Non-Veg, and level 2: 1 = Terrestrial, 2 = Aquatic
Code	ID	Name	Land cover (LC) Class Definition	ID
11	OW	Open water	OW: Areas of open water, generally with less than 25% cover of vegetation or soil	B4—Non-vegetated aquatic
12	PIS	Perennial Ice/Snow	PIS: Areas characterized by a perennial cover of ice and/or snow, generally greater than 25% of total cover.	B4
21 22 23 24	DOS DLI DMI DHI	Developed, Open Space Developed, Low Intensity Developed, Medium Intensity Developed, High Intensity	DOS: Includes areas with a mixture of some constructed materials, but mostly vegetation in the form of lawn grasses. Impervious surfaces account for less than 20 percent of total cover. These areas most commonly include large-lot single-family housing units, parks, golf courses, and vegetation planted in developed settings for recreation, erosion control, or aesthetic purposes. DLI, DMI, DHI: refer to the “National Land Cover Database 2006 (NLCD2006),” Multi-Resolution Land Characteristics Consortium (MRLC), 2013.	B3—Non-vegetated terrestrial/A1—Vegetated terrestrial
31	BL	Barren Land (Rock/Sand/Clay)	BL: Barren areas of bedrock, desert pavement, scarps, talus, slides, volcanic material, glacial debris, sand dunes, strip mines, gravel pits and other accumulations of earthen material. Generally, vegetation accounts for less than 15% of total cover. As a consequence of this constraint, class BL covers only 1.21% of the CONUS total surface.	B3
41 42 43	DF EF MF	Deciduous Forest Evergreen Forest Mixed Forest	DF: Areas dominated by trees generally greater than 5 m tall, and greater than 20% of total vegetation cover. More than 75 percent of the tree species shed foliage simultaneously in response to seasonal change. EF: Areas dominated by trees generally greater than 5 m tall, and greater than 20% of total vegetation cover. More than 75 percent of the tree species maintain their leaves all year. Canopy is never without green foliage. MF: Mixed Forest—Areas dominated by trees generally greater than 5 m tall, and greater than 20% of total vegetation cover. Neither deciduous nor evergreen species are greater than 75 percent of total tree cover.	A1
51 52	-SS	Dwarf Scrub ^2^ Scrub/Shrub	SS: Areas dominated by shrubs; less than 5 m tall with shrub canopy typically greater than 20% of total vegetation. This class includes true shrubs, young trees in an early successional stage or trees stunted from environmental conditions. The aforementioned definition of class BL means that class SS may feature a vegetated cover which accounts for 15% of total cover or more.	A1/B3
71 72 73 74	GH---	Grassland/Herbaceous Sedge Herbaceous ^2^ Lichens ^2^ Moss ^2^	GH: Areas dominated by grammanoid or herbaceous vegetation, generally greater than 80% of total vegetation. These areas are not subject to intensive management such as tilling, but can be utilized for grazing. The aforementioned definition of class BL means that class GH may feature a vegetated cover which accounts for 15% of total cover or more.	A1/B3
81 82	PH CC	Pasture/Hay Cultivated Crops	PH: Areas of grasses, legumes, or grass-legume mixtures planted for livestock grazing or the production of seed or hay crops, typically on a perennial cycle. Pasture/hay vegetation accounts for greater than 20 percent of total vegetation. CC: Areas used for the production of annual crops, such as corn, soybeans, vegetables, tobacco, and cotton, and also perennial woody crops such as orchards and vineyards. Crop vegetation accounts for greater than 20% of total vegetation. This class also includes all land being actively tilled.	A1
90 95	WW EHW	Woody Wetlands Emergent Herbaceous Wetland	WW: Areas where forest or shrubland vegetation accounts for greater than 20 percent of vegetative cover and the soil or substrate is periodically saturated with or covered with water. EHW: Areas where perennial herbaceous vegetation accounts for greater than 80% of vegetative cover and the soil or substrate is periodically saturated with or covered with water.	A2—Vegetated aquatic

For further details, refer to the “National Land Cover Database 2006 (NLCD2006),” Multi-Resolution Land Characteristics Consortium (MRLC), 2013. The right column instantiates a possible binary relationship R: A ⇒ B ⊆ A × B from set A = NLCD legend to set B = 2-level 4-class Dichotomous Phase (DP) taxonomy of the Food and Agriculture Organization of the United Nations (FAO)—Land Cover Classification System (LCCS) (Di Gregorio & Jansen, 2000), refer to Figure 3.

In this experimental framework, the test SIAM-WELD annual color map time-series for the years 2006–2009 and the reference USGS NLCD 2006 map share the same spatial extent and spatial resolution, but their map legends are not the same. These working hypotheses are neither trivial nor conventional in the RS literature, where thematic map quality assessment strategies typically adopt an either random or non-random sampling strategy and assume that the test and reference thematic map dictionaries coincide (Stehman & Czaplewski, 1998). Starting from a stratified random sampling protocol presented in Baraldi et al. (2014), the secondary contribution of the present study was to develop a novel protocol for wall-to-wall comparison without sampling of two thematic maps featuring the same spatial extent and spatial resolution, but whose legends can differ.

For the sake of readability this paper is split into two, the present Part 1—Theory and the subsequent Part 2—Validation. An expert reader familiar with static color naming in cognitive science, spanning from linguistics to human vision and CV, can skip the present Part 1, either totally or in part. To make this paper self-contained and provided with a relevant survey value, the Part 1 is organized as follows. The multidisciplinary background of color naming is discussed in Chapter 2. Chapter 3 reviews the long history of prior knowledge-based decision trees for MS color naming presented in the RS literature. To cope with thematic map legends that do not coincide and must be harmonized (reconciled, associated, translated) (Ahlqvist, 2005), such as dictionaries of MS color names in the image-domain and LC class names in the scene-domain, Chapter 3 proposes an original hybrid inference guideline to identify a categorical variable-pair relationship, where prior beliefs are combined with additional evidence inferred from new data. An original measure of categorical variable-pair association (harmonization) in a binary relationship is proposed in Chapter 4. In the subsequent Part 2, GEO-CEOS stage 4 *Val* results are collected by an original protocol for wall-to-wall thematic map quality assessment without sampling, where legends of the test SIAM-WELD annual map time-series and reference USGS NLCD 2006 map are harmonized. Conclusions are that the annual SIAM-WELD map time-series for the years 2006–2009 provides a first example of GEO-CEOS stage 4 *validated* ESA EO Level 2 SCM product, where the Level 2 SCM legend is the “augmented” 2-level 4-class FAO LCCS taxonomy at the DP Level 1 (vegetation/non-vegetation) and DP Level 2 (terrestrial/aquatic), added with extra class “rest of the world”.

## 2. Problem background of color naming in cognitive science

Within the cognitive science domain, vision is synonym of scene-from-image reconstruction and understanding, see Figure 13. Encompassing both biological vision and CV, vision is a cognitive (*information-as-data-interpretation*) problem inherently ill-posed in the Hadamard sense (Hadamard, 1902); hence, it is very difficult to solve. Vision is non-polynomial (NP)-hard in computational complexity (Frintrop, 2011; Tsotsos, 1990) and requires *a priori* knowledge in addition to sensory data to become better posed for numerical solution (Cherkassky & Mulier, 1998). It is inherently ill-posed because affected by, first, data dimensionality reduction from the 4D spatio-temporal scene-domain to the (2D) image-domain and, second, by a semantic information gap from ever-varying sensations in the (2D) image-domain to stable percepts in the mental model of the 4D scene-domain (Matsuyama & Hwang, 1990). On the one hand, ever-varying sensations collected from the 4D spatio-temporal physical world are synonym of observables, numeric/quantitative variables of sub-symbolic value or sensory data provided with a physical unit of measure, such as TOARF or SURF values, but featuring no semantics corresponding to abstract concepts, like perceptual categories or mental states. On the other hand, in a modeled world, also known as world ontology, mental world or “world model” (Matsuyama & Hwang, 1990), stable percepts are nominal/categorical/qualitative variables of symbolic value, i.e., they are categorical variables provided with semantics, such as LC class names belonging to a hierarchical FAO LCCS taxonomy of the world (Di Gregorio & Jansen, 2000), see Figure 3.

**Figure 14.**
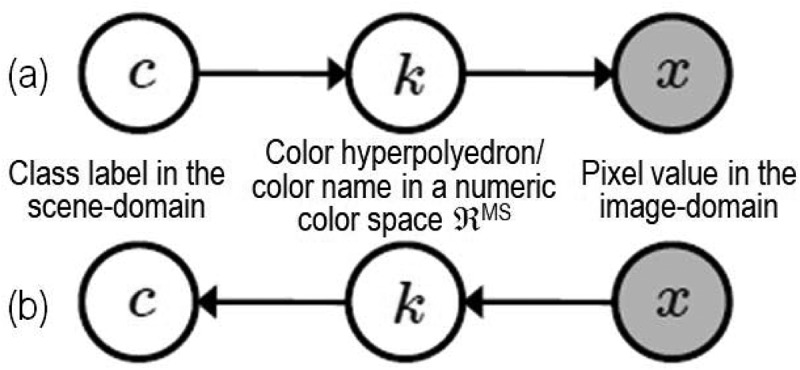
Graphical model of color naming, adapted from Shotton et al. (2009). Let us consider x as a (sub-symbolic) numeric variable, such as MS color values of a population of spatial units, with x ∈ ℜ^MS^, while c represents a categorical variable of symbolic classes in the physical world, with c = 1, …, ObjectClassLegendCardinality. (a) According to Bayesian theory, posterior probability p(c|x) ∝ p(x|c)p(c) = p(c) $\overset{ColorVocabularyCardinality}{\underset{ColorName=k=1}{\sum }}p\left(x|k\right)p\left(k|c\right)$, where color names, equivalent to color hyperpolyhedra in a numeric color space ℜ^MS^, provide a partition of the domain of change, ℜ^MS^, of numeric variable x. (b) For discriminative inference, the arrows in the graphical model are reversed using Bayes rule. Hence, a vocabulary of color names, physically equivalent to a partition of a numeric color space ℜ^MS^ into color name-specific hyperpolyhedra, is conceptually equivalent to a latent/hidden/hypothetical variable linking observables (sub-symbolic sensory data) in the real world, specifically, color values, to a categorical variable of semantic (symbolic) quality in the mental model of the physical world (world ontology, world model).

In statistics, the popular concept of latent/hidden variables was introduced to fill the information gap from input observables to target categorical variables. Latent/hidden variables are not directly measured, but inferred from observable numeric variables to link sensory data in the real world to categorical variables of semantic quality in the modeled world. “The terms hypothetical variable or hypothetical construct may be used when latent variables correspond to abstract concepts, like perceptual categories or mental states” (Baraldi, 2017; Shotton, Winn, Rother, & Criminisi, 2009; Wikipedia, 2018b). Hence, to fill the semantic gap from low-level numeric variables of sub-symbolic quality to high-level categorical variables of semantic value, hypothetical variables, such as categorical BC names (Benavente et al., 2008; Berlin & Kay, 1969; Gevers, Gijsenij, van de Weijer, & Geusebroek, 2012; Griffin, 2006), are expected to be mid-level categorical variables of “semi-symbolic” quality, i.e., hypothetical variables are nominal variables provided with a semantic value located “low” in a hierarchical ontology of the world, such as the hierarchical FAO LCCS taxonomy (Di Gregorio & Jansen, 2000), but always superior to zero, where zero is the semantic value of sub-symbolic numeric variables, see Figure 14.

In vision, spatial topological and spatial non-topological information components typically dominate color information (Baraldi, 2017; Matsuyama & Hwang, 1990). This thesis is proved by the undisputable fact that achromatic (panchromatic) human vision, familiar to everybody when wearing sunglasses, is nearly as effective as chromatic vision in scene-from-image reconstruction and understanding. Driven from perceptual evidence in human vision typically investigated by cognitive science, see Figure 13, a necessary not sufficient condition for a CV system to prove it fully exploits spatial topological and spatial non-topological information components in addition to color is to perform nearly the same when input with either panchromatic or color imagery. Stemming from *a priori* knowledge of human vision available in addition to sensory data, this necessary not sufficient condition can be adopted to make an inherently ill-posed CV system design and implementation problem better constrained for numerical solution.

Deeply investigated in CV (Frintrop, 2011; Sonka et al., 1994), content-based image retrieval (Smeulders, Worring, Santini, Gupta, & Jain, 2000) and EO image applications proposed by the RS community (Baraldi, 2017; Baraldi & Boschetti, 2012a, 2012b; Matsuyama & Hwang, 1990; Nagao & Matsuyama, 1980; Shackelford & Davis, 2003a, 2003b), well-known visual features are: (i) color values, typically discretized by humans into a finite and discrete vocabulary of BC names (Benavente et al., 2008; Berlin & Kay, 1969; Gevers et al., 2012; Griffin, 2006); (ii) local shape (Baraldi, 2017; Wenwen Li et al. 2013); (iii) texture, defined as the perceptual spatial grouping of texture elements known as texels (Baraldi, 2017; Julesz, 1986; Julesz et al., 1973) or tokens (Marr, 1982); (iv) inter-object spatial topological relationships, e.g., adjacency, inclusion, etc., and (v) inter-object spatial non-topological relationships, e.g., spatial distance, angle measure, etc. In vision, color is the sole visual property available at the imaging sensor’s spatial resolution, i.e., at pixel level. In other words, pixel-based information is spatial context independent. i.e., per-pixel information is exclusively related to color properties. Among the aforementioned visual variables, per-pixel color values are the sole non-spatial (spatial context-insensitive) numeric variable.

**Figure 15.**
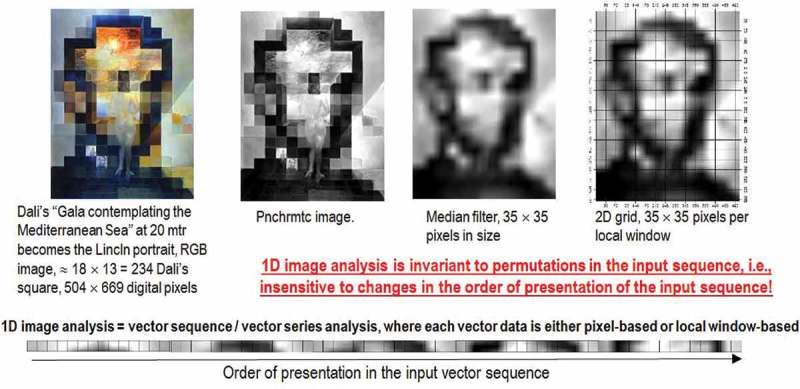
Example of 1D image analysis. Synonym of 1D analysis of a 2D gridded dataset, it is affected by spatial data dimensionality reduction. The (2D) image at left is transformed into the 1D vector data stream shown at bottom, where vector data are either pixel-based or spatial context-sensitive, e.g., local window-based. This 1D vector data stream, either pixel-based or local window-based, means nothing to a human photointerpreter. When it is input to a traditional inductive data learning classifier, this 1D vector data stream is what the inductive classifier actually sees when watching the (2D) image at left. Undoubtedly, computers are more successful than humans in 1D image analysis, invariant to permutations in the input vector data sequence, such as in orderless pooling encoders (Cimpoi et al., 2014). Nonetheless, humans are still far more successful than computers in 2D image analysis, synonym of spatial topology-preserving (retinotopic) image analysis (Tsotsos, 1990), sensitive to permutations in the input vector data sequence, such as in order-sensitive pooling encoders (Cimpoi et al., 2014).

Neglecting the fact that spatial topological and spatial non-topological information components typically dominate color information in both the (2D) image-domain and the 4D spatio-temporal scene-domain involved with the cognitive task of vision (Matsuyama & Hwang, 1990), traditional EO-IUSs adopt a 1D image analysis approach, see Figure 15. In 1D image analysis, a 1D streamline of vector data, either spatial context-sensitive (e.g., window-based or image object-based like in OBIA approaches) or spatial context-insensitive (pixel-based), is processed insensitive to changes in the order of presentation of the input sequence. In practice 1D image analysis is invariant to permutations, such as in orderless pooling encoders (Cimpoi et al., 2014). When vector data are spatial context-sensitive then 1D image analysis ignores spatial topological information. When vector data are pixel-based then 1D image analysis ignores both spatial topological and spatial non-topological information components. Prior knowledge-based color naming of a spatial unit x in the image-domain, where x is either (0D) point, (1D) line or (2D) polygon defined according to the OGC nomenclature (OGC, 2015), is a special case of 1D image analysis, either pixel-based or image object-based, where spatial topological and/or spatial non-topological information are ignored, such as in SIAM’s static color naming (Baraldi et al., 2006).

**Figure 16.**
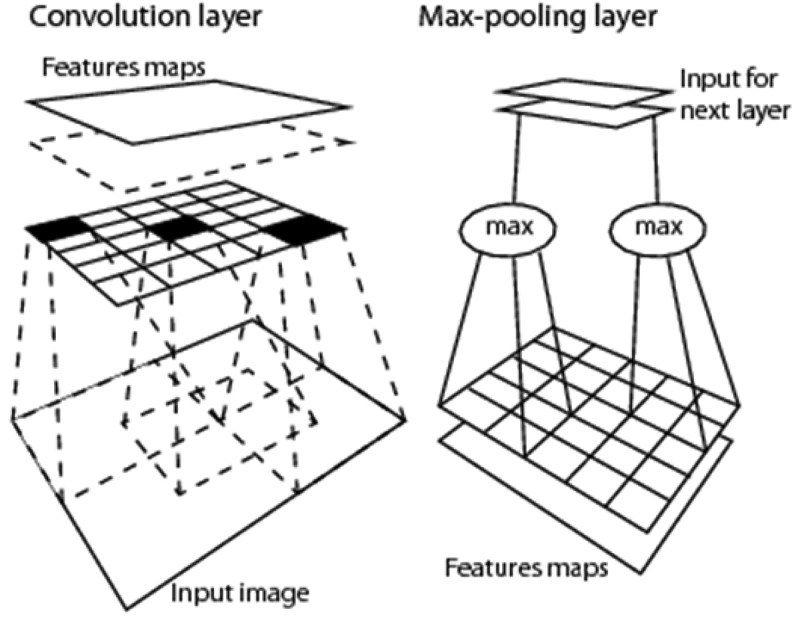
2D image analysis, synonym of spatial topology-preserving (retinotopic) feature mapping in a (2D) image-domain (Tsotsos, 1990). Activation domains of physically adjacent processing units in the 2D array of convolutional spatial filters are spatially adjacent regions in the 2D visual field. Provided with a superior degree of biological plausibility in modeling 2D spatial topological and spatial non-topological information, distributed processing systems capable of 2D image analysis, such as deep convolutional neural networks (DCNNs), typically outperform traditional 1D image analysis approaches. Will computers become as good as humans in 2D image analysis?

Alternative to 1D image analysis, 2D image analysis relies on a sparse (distributed) 2D array (2D regular grid) of local spatial filters, suitable for spatial topology-preserving (retinotopic) feature mapping (DiCarlo, 2017; Fritzke, 1997a; Martinetz, Berkovich, & Schulten, 1994; Tsotsos, 1990), sensitive to permutations in the input vector data sequence, such as in order-sensitive pooling encoders (Cimpoi et al., 2014), see Figure 16. The human brain’s organizing principle is topology-preserving feature mapping (Feldman, 2013). In the biological visual system, topology-preserving feature maps are primarily spatial, where activation domains of physically adjacent processing units in the 2D array of convolutional filters are spatially adjacent regions in the 2D visual field. Provided with a superior degree of biological plausibility in modeling 2D spatial topological and spatial non-topological information components, distributed processing systems capable of 2D image analysis, such as physical model-based (“hand-crafted”) 2D wavelet filter banks (Mallat, 2016) and end-to-end inductive learning-from-data DCNNs, typically outperform 1D image analysis approaches (Cimpoi et al., 2014; DiCarlo, 2017), although DCNNs are the subject of increasing criticisms by the artificial intelligence (AI) community (DiCarlo, 2017; Etzioni, 2017; Marcus, 2018). This apparently trivial consideration is at odd with a relevant portion of the RS literature, where pixel-based 1D image analysis is mainstream, followed in popularity by spatial context-sensitive 1D image analysis implemented within the OBIA paradigm (Blaschke et al., 2014). Undoubtedly, computers are more successful than humans in 1D image analysis, invariant to permutations in the input vector data sequence (Cimpoi et al., 2014). Nonetheless, humans are still far more successful than computers in 2D image analysis, synonym of spatial topology-preserving feature mapping (Tsotsos, 1990), which implies sensitivity to permutations in the input vector data sequence (Cimpoi et al., 2014).

Since traditional EO-IUSs adopt a 1D image analysis approach, where dominant spatial information is omitted either totally or in part in favor of secondary color information, it is useful to turn attention to the multidisciplinary framework of cognitive science to shed light on how humans cope with color information. According to cognitive science which includes linguistics, the study of languages, see Figure 13, humans discretize (fuzzify) ever-varying quantitative (numeric) photometric and spatio-temporal sensations into stable qualitative/categorical/nominal percepts, eligible for use in symbolic human reasoning based on a convergence-of-evidence approach (Matsuyama & Hwang, 1990). In their seminal work, Berlin and Kay proved that 20 human languages, spoken across space and time in the real world, partition quantitative color sensations collected in the visible portion of the electromagnetic spectrum, see Figure 1, onto the same “universal” vocabulary of eleven BC names (Berlin & Kay, 1969): black, white, gray, red, orange, yellow, green, blue, purple, pink and brown. In a 3D monitor-typical red-green-blue (RGB) data cube, BC names are intuitive to think of and easy to visualize. They provide a mutually exclusive and totally exhaustive partition of a monitor-typical RGB data cube into RGB polyhedra neither necessarily connected nor convex, see Figure 7 (Benavente et al., 2008; Griffin, 2006). Since they are community-agreed upon to be used by members of the same community, RGB BC polyhedra are prior knowledge-based, i.e., stereotyped, non-adaptive-to-data (static), general-purpose, application- and user-independent. Multivariate measurement space partitioning into a discrete and finite set of mutually exclusive and totally exhaustive hyperpolyhedra copes with the transformation of a numeric variable into a categorical variable, see Figure 7. Numeric variable discretization is a typical problem in many scientific disciplines, such as inductive VQ in machine learning-from-data (Cherkassky & Mulier, 1998) and deductive numeric variable fuzzification into discrete fuzzy sets, e.g., low, medium and high, in fuzzy logic (Zadeh, 1965), refer to Chapter 1.

To summarize, human languages refer to human colorimetric perception in terms of a stable, prior knowledge-based vocabulary (codebook) of BC names (codewords) non-adaptive to data, physically equivalent to a discrete and finite set of mutually exclusive and totally exhaustive hyperpolyhedra, neither necessarily convex nor connected in a numeric MS color space, identified as ℜ^MS^, where MS >2, e.g., MS = 3 like in a monitor-typical RGB data cube, see Figure 7. These BC names are conceptually equivalent to a latent/hypothetical categorical variable of semi-symbolic quality, see Figure 14, capable of linking sub-symbolic sensory data in the real world, specifically color values in color space ℜ^MS^, to categorical variables of semantic (symbolic) quality in the world model, also known as world ontology or mental world, made of abstract concepts, like perceptual categories of real-world objects or mental states.

In an analytic model of vision based on a convergence-of-evidence approach, the first original contribution of the present Part 1 is to encode prior knowledge about color naming into a CV system by design, as described hereafter. Irrespective of their Pearson inter-feature cross-correlation, if any, it is easy to prove that individual sources of visual evidence, such as color, local shape, texture and inter-object spatial relationships, are statistically independent because, in general, Pearson’s linear cross-correlation does not imply causation (Baraldi, 2017; Baraldi & Soares, 2017; Pearl, 2009). According to a “naive” hypothesis of conditional independence of visual features color, local shape, texture and inter-object spatial relationships, when target classes of observed objects in the real-world scene are c = 1, …, ObjectClassLegendCardinality, for a given discrete spatial unit x in the image-domain, either 0D point, 1D line or 2D polygon (OGC, 2015), then the well-known “naïve” Bayes classification formulation (Bishop, 1995) becomes

(1) $$\begin{array}{l} \text{p}\left(\text{c|}\text{ColorValue}\left(\text{x}\right),\text{ShapeValue}\left(\text{x}\right),\text{TextureValue}\left(\text{x}\right),\text{SpatialRelationships}\left(\text{x},\text{Neigh}\left(\text{x}\right)\right)\right)\\ \text{=p}\left({\text{c|F}}_{\text{i}},\ldots ,{\text{F}}_{\text{I=4}}\right)\text{=p}\left(\text{c}\right)\prod_{i=1}^{I=4}\text{p}\left({\text{F}}_{\text{i}}\text{|c}\right)\text{=p}\left(\text{c}\right)•\text{p}\left(\text{ColorValue}\left(\text{x}\right)\text{|c}\right)•\text{p}\left(\text{ShapeValue}\left(\text{x}\right)\text{|c}\right)\\ •\text{p}\left(\text{TextureValue}\left(\text{x}\right)\text{|c}\right)•\text{p}\left(\text{SpatialRelationships}\left(\text{x},\text{Neigh}\left(\text{x}\right)\right)\text{|c}\right)\leq \text{min}\{\text{p}\left(\text{c|}\text{ColorValue}\left(\text{x}\right)\right),\\ \text{p}(\text{c|}\text{ShapeValue}\left(\text{x}\right)),\text{p}\left(\text{c|}\text{TextureValue}\left(\text{x}\right)\right),\text{p}\left(\text{c|}\text{SpatialRelationships}\left(\text{x},\text{Neigh}\left(\text{x}\right)\right)\right)\},\text{c}\text{=1},\ldots ,\text{ObjectClassLegendCardinality} \end{array}$$

where ColorValue(x) belongs to a MS measurement space ℜ^MS^, i.e., ColorValue(x) ∈ ℜ^MS^, and Neigh(x) is a generic 2D spatial neighborhood of spatial unit x in the (2D) image-domain. Equation (1) shows that any convergence-of-evidence approach is more selective than each individual source of evidence, in line with a focus-of-visual attention mechanism (Frintrop, 2011). For the sake of simplicity, if priors are ignored because considered equiprobable in a maximum class-conditional likelihood inference approach alternative to a maximum a posteriori optimization criterion, then Equation (1) becomes

(2) $$\begin{array}{rl} & p\left(c|\;ColorValue\left(x\right),ShapeValue\left(x\right),TextureValue\left(x\right),SpatialRelationships\left(x,Neigh\left(x\right)\right)\right)\;\propto \\ & \;\;\;\;\;\;\;\;p\left(ColorValue\left(x\right)|\;c\right)\;∙\;p\left(ShapeValue\left(x\right)|\;c\right)\;∙\;p\left(TextureValue\left(x\right)|\;c\right)\;∙\;p(SpatialRelationships\\ & \;\;\;\;\;\;\;\;\;\left(x,Neigh\left(x\right)\right)|\;c)=[\sum_{ColorName=1}^{ColorVocabularyCardinality}p\left(ColorValue\left(x\right)|ColorName\right)p\left(ColorName|c\right)]\;∙\;\\ & \;\;\;\;\;\;\;\;\;\;p\left(ShapeValue\left(x\right)|\;c\right)\;p\left(TextureValue\left(x\right)|c\right)\;∙\;p\left(SpatialRelationships\left(x,Neigh\left(x\right)\right)|\;c\right)c\\ & \;\;\;\;\;\;\;\;\;=1,\ldots ,ObjectClassLegendCardinality \end{array}$$

where color space ℜ^MS^ is partitioned into hyperpolyhedra, equivalent to a discrete and finite vocabulary of static color names, with ColorName = 1, …, ColorVocabularityCardinality. To further simplify Equation (2), its canonical interpretation based on frequentist statistics can be relaxed by fuzzy logic (Zadeh, 1965), so that the logical-AND operator is replaced by a fuzzy-AND (min) operator, inductive class-conditional probability p(x| c) ∈ [0, 1], where $\overset{ObjectClassLegendCardinality}{\underset{c=1}{\sum }}p\left(x|c\right)\;>=0$, is replaced by a deductive membership (compatibility) function m(x| c) ∈ [0, 1], where $\overset{ObjectClassLegendCardinality}{\underset{c=1}{\sum }}m\left(x|c\right)\;>=0$ according to the principles of fuzzy logic, where compatibility/membership does not mean probability, and color space hyperpolyhedra are considered mutually exclusive and totally exhaustive. If these simplifications are adopted, then Equation (2) becomes

(3) $$\begin{array}{rl} & m\left(c|\;ColorValue\left(x\right),ShapeValue\left(x\right),TextureValue\left(x\right),SpatialRelationships\left(x,Neigh\left(x\right)\right)\right)\;\propto \;min\\ & \;\;\;\;\;\;\;\;\;\;{\{\sum }_{ColorName=1}^{ColorVocabularyCardinality}m\left(ColorValue\left(x\right)|\;ColorName\right)m\left(ColorName|c\right),m\left(ShapeValue\left(x\right)|\;c\right),\\ & \;\;\;\;\;\;\;\;\;m\left(TextureValue\left(x\right)|\;c\right),m\left(SpatialRelationships\left(x,Neigh\left(x\right)\right)|\;c\right)\}=min\{m\left(ColorName^{*}|\;c\right),\\ & \;\;\;\;\;\;\;\;\;m\left(ShapeValue\left(x\right)|\;c\right),m\left(TextureValue\left(x\right)|\;c\right),m\left(SpatialRelationships\left(x,Neigh\left(x\right)\right)|\;c\right)\},c=1,\ldots ,\\ & \;\;\;\;\;\;\;\;\;ObjectClassLegendCardinality,where\;ColorName^{*}\;\in \;\left\{1,ColorVocabularyCardinality\right\},such\;that\\ & \;\;\;\;\;\;\;\;\;\;m\left(ColorValue\left(x\right)|ColorName^{*}\right)=1\;and\;m\left(ColorName^{*}|\;c\right)\in \left\{0,1\right\}. \end{array}$$

In Equation (3), the following considerations hold.

- Each numeric ColorValue(x) in color space ℜ^MS^ belongs to a single color name (hyperpolyhedron) ColorName* in the static color name vocabulary, i.e., ∀ ColorValue(x) ∈ ℜ^MS^, $\overset{ColorVocabularyCardinality}{\underset{ColorName=1}{\sum }}m\left(ColorValue\left(x\right)|ColorName\right)=m\left(ColorValue\left(x\right)|ColorName^{*}\right)=1$ holds, where m(ColorValue(x)| ColorName) ∈ {0, 1} is a binary (crisp, hard) membership function, with ColorName = 1, …, ColorVocabularyCardinality and ColorName* ∈ {1, ColorVocabularyCardinality}.
- Set A = VocabularyOfColorNames, with cardinality |A| = a = ColorVocabularyCardinality, and set B = LegendOfObjectClassNames, with cardinality |B| = b = ObjectClassLegendCardinality, can be considered a bivariate categorical random variable where two univariate categorical variables A and B are generated from a single population. A binary relationship from set A to set B, R: A ⇒ B, is a subset of the 2-fold Cartesian product (product set) A × B, whose size is rows × columns = a × b, hence, R: A ⇒ B ⊆ A × B. The Cartesian product of two sets A × B is a set whose elements are ordered pairs. Hence, the Cartesian product is non-commutative, A × B ≠ B × A. In agreement with common sense, see Table 3, binary relationship R: VocabularyOfColorNames ⇒ LegendOfObjectClassNames is a set of ordered pairs where each ColorName can be assigned to none, one or several classes of observed scene-objects with class index c = 1, …, ObjectClassLegendCardinality, whereas each class of observed objects can be assigned with none, one or several color names to define the class-specific colorimetric attribute. Binary membership values m(ColorName| c) ∈ {0, 1} and m(c| ColorName) ∈ {0, 1}, with c = 1, …, ObjectClassLegendCardinality and ColorName = 1, …, ColorVocabularyCardinality, can be community-agreed upon based on various kinds of evidence, whether viewed all at once or over time, such as a combination of prior beliefs with additional evidence inferred from new data in agreement with a Bayesian updating rule, largely applied in AI, including design and implementation of expert systems. In Bayesian updating, Bayesian inference is applied iteratively (Ghahramani, 2011; Wikipedia, 2018c): after observing some evidence, the resulting posterior probability can be treated as a prior probability and a new posterior probability computed from new evidence. A binary relationship R: A ⇒ B ⊆ A × B, where sets A and B are categorical variables generated from a single population, guides the interpretation process of a two-way *contingency table*, also known as association matrix, cross tabulation, bivariate table or bivariate frequency table (BIVRFTAB) (Kuzera & Pontius, 2008; Pontius & Connors, 2006; Pontius & Millones, 2011), such that BIVRFTAB = FrequencyCount(A × B). In the conventional domain of frequentist inference with no reference to prior beliefs, a BIVRFTAB is the 2-fold Cartesian product A × B instantiated by the bivariate frequency counts of the two univariate categorical variables A and B generated from a single population. Hence, binary relationship R: A ⇒ B ⊆ A × B ≠ FrequencyCount(A × B) = BIVRFTAB, where one instantiation of the former guides the interpretation process of the latter. In greater detail, for any BIVRFTAB instance, either square or non-square, there is a binary relationship R: A ⇒ B ⊆ A × B that guides the interpretation process, where “correct” binary entry-pair cells of the 2-fold Cartesian product A × B are equal to 1 and located either off-diagonal (scattered) or on-diagonal, if a main diagonal exists when the BIVRFTAB is square. When a BIVRFTAB is estimated from a geospatial population with or without sampling, it is called *overlapping area matrix* (OAMTRX) (Baraldi et al., 2014; Baraldi, Bruzzone, & Blonda, 2005; Baraldi et al., 2006; Beauchemin & Thomson, 1997; Lunetta & Elvidge, 1999; Ortiz & Oliver, 2006; Pontius & Connors, 2006). When the binary relationship R: A ⇒ B is a bijective function (both 1–1 and onto), i.e., when the two categorical variables A and B estimated from a single population coincide, then the BIVRFTAB instantiation is square and sorted; it is typically called confusion matrix (CMTRX) or error matrix (Congalton & Green, 1999; Lunetta & Elvidge, 1999; Pontius & Millones, 2011; Stehman & Czaplewski, 1998). In a CMTRX, the main diagonal guides the interpretation process. For example, a square OAMTRX = FrequencyCount(A × B), where A = test thematic map legend, B = reference thematic map legend such that cardinality a = b, is a CMTRX if and only if A = B, i.e., if the test and reference codebooks are the same sorted set of concepts or categories. In general the class of (square and sorted) CMTRX instances is a special case of the class of OAMTRX instances, either square or non-square, i.e., OAMTRX ⊃ CMTRX. A similar consideration holds about summary Q^2^Is generated from an OAMTRX or a CMTRX, i.e., Q^2^I(OAMTRX) ⊃ Q^2^I(CMTRX) (Baraldi et al., 2014, 2005, 2006).

**Table 3.** Example of a binary relationship R: A ⇒ B ⊆ A × B from set A = VocabularyOfColorNames, with cardinality |A| = a = ColorVocabularyCardinality = 11, and the set B = LegendOfObjectClassNames, with cardinality |B| = b = ObjectClassLegendCardinality = 3

			Target classes of individuals (entities in a conceptual model for knowledge representation built upon an ontology language)		
			Class 1, Water body	Class 2, Tulip flower	Class 3, Italian tile roof
Color names	Black			√	
	Blue		√	√	
	Brown		√	√	√
	Grey				
	Green		√	√	
	Orange			√	
	Pink			√	
	Purple			√	
	Red			√	√
	White			√	
	Yellow			√	

The latter dictionary is a superset of the typical taxonomy of land cover (LC) classes adopted by the RS community. “Correct” entry-pairs (marked with √) must be: (i) selected by domain experts based on a hybrid combination of deductive prior beliefs with inductive evidence from data, refer to Table 5, and (ii) community-agreed upon

**Table 4.** Rule set (structural knowledge) and order of presentation of the rule set (procedural knowledge) adopted by the prior knowledge-based MS reflectance space quantizer called Spectral Classification of surface reflectance signatures (SPECL), implemented within the ATCOR commercial software toolbox (Dorigo et al., 2009; Richter & Schläpfer, 2012a, 2012b)

Index	Spectral Categories	Spectral Rule (based on reflectance measured at Landsat TM central wave bands: b1 is located at 0.48 μm, b2 at 0.56 μm, b3 at 0.66 μm, b4 at 0.83 μm, b5 at 1.6 μm, b7 at 2.2 μm)	Pseudo-color
1	Snow/ice	b4/b3 ≤ 1.3 AND b3 ≥ 0.2 AND b5 ≤ 0.12	
2	Cloud	b4 ≥ 0.25 AND 0.85 ≤ b1/b4 ≤ 1.15 AND b4/b5 ≥ 0.9 AND b5 ≥ 0.2	
3	Bright bare soil/sand/cloud	b4 ≥ 0.15 AND 1.3 ≤ b4/b3 ≤ 3.0	
4	Dark bare soil	b4 ≥ 0.15 AND 1.3 ≤ b4/b3 ≤ 3.0 AND b2 ≤ 0.10	
5	Average vegetation	b4/b3 ≥ 3.0 AND (b2/b3 ≥ 0.8 OR b3 ≤ 0.15) AND 0.28 ≤ b4 ≤ 0.45	
6	Bright vegetation	b4/b3 ≥ 3.0 AND (b2/b3 ≥ 0.8 OR b3 ≤ 0.15) AND b4 ≥ 0.45	
7	Dark vegetation	b4/b3 ≥ 3.0 AND (b2/b3 ≥ 0.8 OR b3 ≤ 0.15) AND b3 ≤ 0.08 AND b4 ≤ 0.28	
8	Yellow vegetation	b4/b3 ≥ 2.0 AND b2 ≥_b3 AND b3 ≥ 8.0 AND b4/b5 ≥ 1.5 ^a^	
9	Mix of vegetation/soil	2.0 ≤ b4/b3 ≤ 3.0 AND 0.05 ≤ b3 ≤ 0.15 AND b4 ≥ 0.15	
10	Asphalt/dark sand	b4/b3 ≤ 1.6 AND 0.05 ≤ b3 ≤ 0.20 AND 0.05 ≤ b4 ≤ 0.20^a^ AND 0.05 ≤ b5 ≤ 0.25 AND b5/b4 ≥ 0.7^a^	
11	Sand/bare soil/cloud	b4/b3 ≤ 2.0 AND b4 ≥ 0.15 AND b5 ≥ 0.15^a^	
12	Bright sand/bare soil/cloud	b4/b3 ≤ 2.0 AND b4 ≥ 0.15 AND (b4 ≥ 0.25b OR b5 ≥ 0.30^b^)	
13	Dry vegetation/soil	(1.7 ≤ b4/b3 ≤ 2.0 AND b4 ≥ 0.25^c^) OR (1.4 ≤ b4/b3 ≤ 2.0 AND b7/b5 ≤ 0.83^c^)	
14	Sparse veg./soil	(1.4 ≤ b4/b3 ≤ 1.7 AND b4 ≥ 0.25^c^) OR (1.4 ≤ b4/b3 ≤ 2.0 AND b7/b5 ≤ 0.83 AND b5/b4 ≥ 1.2^c^)	
15	Turbid water	b4 ≤ 0.11 AND b5 ≤ 0.05^a^	
16	Clear water	b4 ≤ 0.02 AND b5 ≤ 0.02^a^	
17	Clear water over sand	b3 ≥ 0.02 AND b3 ≥ b4 + 0.005 AND b5 ≤ 0.02^a^	
18	Shadow		
19	Not classified (outliers)		

^a^ These expressions are optional and only used if b5 is present. ^b^ Decision rule depends on presence of b5. ^c^ Decision rule depends on presence of b7.

**Table 5.** 8-step guideline for best practice in the identification of a dictionary-pair binary relationship based on a hybrid combination of (top-down) prior beliefs, if any, with (bottom-up) frequentist inference

**STEP 1. Dictionary-pair relationship, multivariate occurrence distributions**						
		***Reference Classification (RC)***				
		EvergreenF	DeciduousF	Others		
***Test Classification (TC)***	Vegetation	10	30	60	100	
	Cloud	2	0	10	12	
	Unknowns	0	5	100	105	Tot.
		12	35	170		217
**STEP 2. Dictionary-pair relationship, multivariate probability distributions**						
		***Reference Classification (RC)***				
		EvergreenF	DeciduousF	Others		
***Test Classification (TC)***	Vegetation	0.046082949	0.138248848	0.276498	0.460829	
	Cloud	0.00921659	0	0.046083	0.0553	
	Unknowns	0	0.023041475	0.460829	0.483871	Tot.
		0.055299539	0.161290323	0.78341		1
**STEP 3. Dictionary-pair relationship, cond. prob. (RC|TC)**						
		***Reference Classification (RC)***				
		EvergreenF	DeciduousF	Others		
***Test Classification (TC)***	Vegetation	0.1	0.3	0.6	1	
	Cloud	0.166666667	0	0.833333	1	
	Unknowns	0	0.047619048	0.952381	1	
**STEP 4. Crisp membership function (RC|TC) > TH1 = 0.09.**						
		***Reference Classification (RC)***				
		EvergreenF	DeciduousF	Others		
***Test Classification (TC)***	Vegetation	1	1	1		
	Cloud	1	0	1		
	Unknowns	0	0	1		
**STEP 5. Dictionary-pair relationship, cond. prob. (TC|RC)**						
		***Reference Classification (RC)***				
		EvergreenF	DeciduousF	Others		
***Test Classification (TC)***	Vegetation	0.833333333	0.857142857	0.352941		
	Cloud	0.166666667	0	0.058824		
	Unknowns	0	0.142857143	0.588235		
		1	1	1		
**STEP 6. Crisp membership function (TC|RC) > TH2 = 0.06 ≤ TH1 = 0.09.**						
		***Reference Classification (RC)***				
		EvergreenF	DeciduousF	Others		
***Test Classification (TC)***	Vegetation	1	1	1		
	Cloud	1	0	0		
	Unknowns	0	1	1		
**STEP 7. OR{Crisp membership function (TC|RC), Crisp membership function(RC|TC)}**						
		***Reference Classification (RC)***				
		EvergreenF	DeciduousF	Others		
***Test Classification (TC)***	Vegetation	1	1	1		
	Cloud	1	0	1		
	Unknowns	0	1	1		
**STEP 8. Top-down (driven-by-prior knowledge) scrutiny of bottom-up (data-driven)**						
**“temporary correct” or “temporary non-correct” cells**						
		***Reference Classification (RC)***				
		EvergreenF	DeciduousF	Others		
***Test Classification (TC)***	Vegetation	1	1	1		
	Cloud	0	0	1		
	Unknowns	0	0	1		

Equation (3) shows that for any spatial unit x in the image-domain, when a hierarchical CV classification approach estimates posterior m(c| ColorValue(x), ShapeValue(x), TextureValue(x), SpatialRelationships(x, Neigh(x))) starting from an *a priori* knowledge-based near real-time color naming first stage, where condition m(ColorValue(x)| ColorName*) = 1 holds, if condition m(ColorName*| c) = 0 is true according to a static community-agreed binary relationship R: VocabularyOfColorNames ⇒ LegendOfObjectClassNames (and vice versa) known *a priori*, see Table 3, then m(c| ColorValue(x), ShapeValue(x), TextureValue(x), SpatialRelationships(x, Neigh(x))) = 0 irrespective of any second-stage assessment of spatial terms ShapeValue(x), TextureValue(x) and SpatialRelationships(x, Neigh(x)), whose computational model is typically difficult to find and computationally expensive. Intuitively, Equation (3) shows that static color naming of any spatial unit x, either (0D) pixel, (1D) line or (2D) polygon, allows the color-based stratification of unconditional multivariate spatial variables into color class-conditional data distributions, in agreement with the statistic stratification principle (Hunt & Tyrrell, 2012) and the divide-and-conquer (*dividi-et-impera*) problem solving approach (Bishop, 1995; Cherkassky & Mulier, 1998; Lipson, 2007). Well known in statistics, the principle of statistic stratification guarantees that “stratification will always achieve greater precision provided that the strata have been chosen so that members of the same stratum are as similar as possible in respect of the characteristic of interest” (Hunt & Tyrrell, 2012).

Whereas 3D color polyhedra are easy to visualize and intuitive to think of in a true- or false-color RGB data cube, see Figure 7, hyperpolyhedra are difficult to think of and impossible to visualize in a MS reflectance space whose spectral dimensionality MS >3, with spectral channels ranging from visible to thermal portions of the electromagnetic spectrum, see Figure 1. Since it is non-adaptive-to-data, any static hyperpolyhedralization of a MS measurement space must be based on *a priori* physical knowledge available in addition to sensory data. Equivalent to a physical data model, static hyperpolyhedralization of a MS data space requires all spectral channels to be provided with a physical unit of radiometric measure, i.e., MS data must be radiometrically calibrated, in compliance with the GEO-CEOS QA4EO *Cal* requirements (GEO-CEOS, 2010, 2010), refer to Chapter 1.

Noteworthy, sensory data provided with a physical unit of measure can be input to both statistical/inductive and physical/deductive models, including hybrid (combined deductive and inductive) inference systems, refer to Chapter 1. On the contrary, uncalibrated dimensionless sensory data can be input to statistical data models exclusively. Although considered mandatory by the GEO-CEOS QA4EO *Cal* guidelines (GEO-CEOS, 2010) and regarded as a well-known “prerequisite for physical model-based analysis of airborne and satellite sensor measurements in the optical domain” (Schaepman-Strub, Schaepman, Painter, Dangel, & Martonchik, 2006), EO data *Cal* is ignored by relevant portions of the RS literature focusing on statistical EO data analytics, such as supervised learning-from-data function regression and classification (Bishop, 1995; Cherkassky & Mulier, 1998). One consequence is that, to date, statistical model-based EO-IUSs dominate the RS literature as well as commercial EO image processing software toolboxes, which typically consist of overly complicated collections of inherently ill-posed inductive machine learning-from-data algorithms (Bishop, 1995; Cherkassky & Mulier, 1998) to choose from based on heuristics (Baraldi, 2017; Baraldi & Boschetti, 2012a, 2012b). This is in contrast with the hybrid inference framework adopted by all biological cognitive systems (Parisi, 1991). In the words of O. Etzioni, “with all due respect to (machine learning-from-data scientists), thought is not a vector, and AI is not a problem in statistics” (Etzioni, 2017; Marcus, 2018).

## 3. Related works in static MS reflectance space hyperpolyhedralization

In the RS discipline, there is a long history of hybrid EO-IUSs in operating mode, suitable for either low-level EO image enhancement (pre-processing) or high-level EO image understanding (classification), where an *a priori* knowledge-based decision tree for static MS reflectance space hyperpolyhedralization is plugged into the hybrid CV system architecture without *Val* by independent means, in disagreement with the GEO-CEOS QA4EO *Val* requirements (GEO-CEOS, 2010; Group on Earth Observation/Committee on Earth Observation Satellites (GEO-CEOS WGCV, 2015), refer to Chapter 1.

In recent years, the SIAM stratification of single-date MS imagery into MS color names was applied to MS image topographic correction, which is a traditional *chicken-and-egg* dilemma (Bishop & Colby, 2002; Bishop et al., 2003; Riano et al., 2003), synonym of inherently ill-posed problem in the Hadamard sense (Hadamard, 1902). When an inherently ill-posed MS image topographic correction was better conditioned for numeric solution by a prior knowledge-based SIAM color naming (masking) first stage, it required no human-machine interaction to run (Baraldi et al., 2010c), in compliance with process requirements of systematic ESA EO Level 2 product generation (ESA, 2015; DLR & VEGA, 2011; CNES, 2015), see Figure 12.

In the Atmospheric/Topographic Correction for Satellite Imagery (ATCOR) commercial software product, several deductive spectral pixel-based decision trees are implemented for use in different stages of an EO data enhancement pipeline (Baraldi et al., 2013; Baraldi & Humber, 2015; Dorigo et al., 2009; Richter & Schläpfer, 2012a, 2012b; Schläpfer, Richter & Hueni, 2009), see Figure 11. One of the ATCOR’s prior knowledge-based per-pixel decision trees delivers as output a haze/cloud/water (and snow) classification mask file (“image_out_hcw.bsq”). In addition, ATCOR includes a so-called prior knowledge-based decision tree for Spectral Classification of surface reflectance signatures (SPECL) (Baraldi & Humber, 2015; Baraldi et al., 2013; Dorigo et al., 2009), see Table 4. Unfortunately, SPECL has never been tested by its authors in the RS literature, although it has been validated by independent means (Baraldi & Humber, 2015; Baraldi et al., 2013).

Supported by NASA, atmospheric effect removal by the Landsat Ecosystem Disturbance Adaptive Processing System (LEDAPS) project relies on exclusion masks for water, cloud, shadow and snow surface types detected by a simple set of prior knowledge-based spectral decision rules applied per pixel. Quantitative analyses of LEDAPS products led by its authors revealed that these exclusion masks are prone to errors, to be corrected in future LEDAPS releases (Vermote & Saleous, 2007). Unfortunately, to date, in a recent comparison of cloud and cloud-shadow detectors, those implemented in LEDAPS scored low among alternative solutions (Foga et al., 2017).

In the 1980s, to provide an automatic alternative to a visual and subjective assessment of the cloud cover on Advanced Very High Resolution Radiometer (AVHRR) quicklook images in the ESA Earthnet archive, Muirhead and Malkawi developed a simple algorithm to classify daylight AVHRR images on a pixel-by-pixel basis into land, cloud, sea, snow or ice and sunglint, such that the classified quicklook image was presented in appropriate pseudo-colors, e.g., green: land, blue: sea, white: cloud, etc. (Muirhead & Malkawi, 1989).

**Figure 17.**
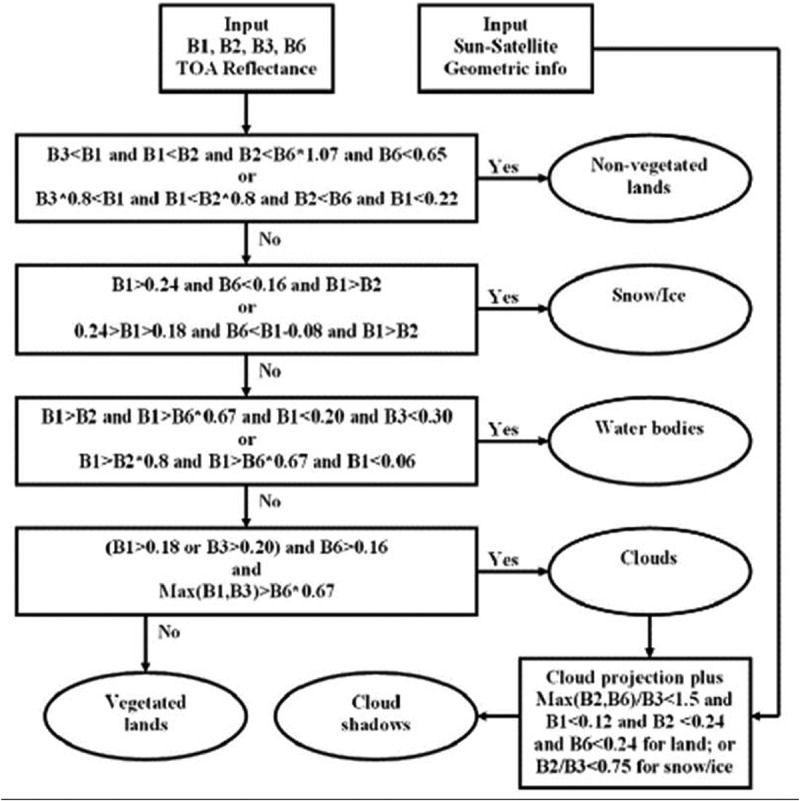
Courtesy of Luo et al. (2008). Canada Centre for Remote Sensing (CCRS)’s flow chart of a physical model-based per-pixel MODIS image classifier integrated into a clear-sky multi-temporal MODIS image compositing system in operating mode. Acronym B stands for MODIS Band.

Developed independently by NASA (Ackerman et al. 1998) and the Canadian Center for Remote Sensing (CCRS) (Luo et al., 2008), pixel-based static decision trees contribute, to date, to the systematic generation of clear-sky Moderate Resolution Imaging Spectroradiometer (MODIS) image composites in operating mode, see Figure 17.

To pursue high-level LC/LCC detection through time, extensions to the time domain of a single-date *a priori* spectral rule base for VQ of an MS reflectance space have become available to the general public in 2015 through the Google Earth Engine (GEE) platform (Simonetti et al. 2015b) or in the form of a commercial LC/LCC map product at national scale (GeoTerraImage, 2015). These are both post-classification approaches for LC/LCC detection based on a time-series of single-date per-pixel prior knowledge-based MS decision-tree classification maps. In practice, MS image time-series analysis in the domain of numeric sub-symbolic variables is replaced by MS color map time-series analysis in the domain of categorical semi-symbolic variables. These two post-classification approaches share the same operational limitations, specifically, they are Landsat sensor series-specific, pixel-based, where spatial topological and spatial non-topological information components are totally ignored, and their post-classification overall accuracy (OA) ∈ [0, 1] is not superior to the product of the single-date classification accuracies in the time-series (Lunetta & Elvidge, 1999). For example,

(4) $$\begin{array}{rl} & Bi\text{-}temporal\;post\text{-}classification\;LC\;change/no\text{-}change\left(LCC\right)detection\;overall\;accuracy\left(OA\right),\\ & \;\;\;\;\;\;OA\text{-}LCC_{1,2}\;\in \;\left[0,1\right],OA\text{-}LCC_{1,2}\;=\;f(OA\;of\;the\;LC\;maps\;at\;time\;T1\;and\;time\;T2,identified\;as\;OA\text{-}LC_{1}\\ & \;\;\;\;\;\;\;and\;OA\text{-}LC_{2}\;respectively)\leq OA\text{-}LC_{1}\times OA\text{-}LC_{2},where\;OA\text{-}LC_{1}\in \left[0,1\right]and\;OA\text{-}LC_{2}\;\in \;\left[0,1\right] \end{array}$$

In Equation (4), if OA-LC_1_ = 0.90 and OA-LC_2_ = 0.90, then OA-LCC_1,2_ ≤ 0.81. Hence, post-classification analysis is recommended for its simplicity if and only if single-date OA values are “high” through the time-series (Baraldi, 2017; Baraldi et al., 2016; Tiede et al., 2016). In other words, a necessary not sufficient pre-condition for multi-temporal image analysis to score “high” in accuracy according to a conceptually simple and computationally efficient post-classification LC change/no-change detection approach is that single-date image classification accuracies score individually “high” through the time-series. The two aforementioned post-classification approaches were both inspired by a year 2006 SIAM instantiation of a static decision tree for Landsat reflectance space hyperpolyhedralization, presented in pseudo-code in the RS literature (Baraldi et al., 2006) and further developed into the SIAM application software available to date (Baraldi, 2017, 2011; Baraldi & Boschetti, 2012a, 2012b; Baraldi et al., 2014, 2010a, 2010b; Baraldi & Humber, 2015; Baraldi et al., 2013; Baraldi, Wassenaar, & Kay, 2010d).

In Boschetti et al. (2015), a year 2013 SIAM instantiation was successfully employed to accomplish post-classification burned area detection in MS image time-series.

Among the aforementioned static decision trees for MS color naming, only SIAM claims scalability to several families of EO imaging sensors featuring different spectral resolutions, see Table 1.

It is obvious but not trivial to emphasize to the RS community that, in human vision and CV, an *a priori* vocabulary of general-purpose data- and application-independent BC names is equivalent to a static sub-symbolic or semi-symbolic categorical variable non-coincident with a symbolic categorical variable whose levels are user- and application-specific classes of objects observed in the 4D spatio-temporal scene-domain, refer to Table 3 and Equation (3). The very same consideration holds for any discrete and finite set of spectral endmembers in mixed pixel analysis, which “cannot always be inverted to unique LC class names” (Adams et al., 1995). It means that spectral endmembers in hyperspectral (HS) image analysis are conceptually equivalent to a static, user- and application-independent vocabulary of BC names, corresponding to a mutually exclusive and totally exhaustive set of neither necessarily convex nor connected hyperpolyhedra in a hyperspectral color space. Whereas the SIAM expert system has been successfully applied to HS imagery for fully automated color naming and superpixel detection (Baraldi, 2017), in the RS literature spectral endmembers detection in HS imagery is traditionally dealt with by inductive learning-from-data algorithms (Ghamisi et al., 2017), which are typically site specific and semiautomatic (Liang, 2004).

Quite surprisingly, the non-coincident assumption between an *a priori* vocabulary of sub-symbolic color names A in the (2D) image-domain and an application-specific legend of symbolic classes B of real-world objects in the 4D scene-domain, where A ≠ B always holds true, refer to Table 3 and Equation (3), appears somehow difficult to acknowledge by relevant portions of the RS community. For example, in the DigitalGlobe Geospatial Big Data platform (GBDX), a patented prior knowledge-based decision tree for pixel-based very high resolution WorldView-2 and WorldView-3 image mapping onto static MS reflectance space hyperpolyhedra (GBDX Registered Name: protogenV2LULC, Provider: GBDX) was proposed to RS end-users as an “Automated Land Cover Classification” (DigitalGlobe, 2016). This program name can be considered somehow misleading because it refers to no EO image mapper of DNs into LC class names, but to a static sub-symbolic color space partitioner, where DNs are mapped onto color name-specific hyperpolyhedra, see Equation (3). Due to the confusion between color names in the (2D) image-domain with target LC classes in the 4D scene-domain, see Table 3, the “Automated Land Cover Classification” computer program is affected by several “known issues” (DigitalGlobe, 2016): “Vegetation: Thin cloud (cloud edges) might be misinterpreted as vegetation; Water: False positives maybe present due to certain types of concrete roofs or shadows; Soils: Ceramic roofing material and some types of asphalt may be misinterpreted as soil,” etc.

In Salmon et al. (2013), a year 2006 SIAM’s *a priori* dictionary of static sub-symbolic MS color names was downscaled in cardinality and sorted in the order of presentation to form a bijective function with a legend of symbolic classes of target objects in the scene-domain. In practice these authors forced a non-square BIVRFTAB to become a (square and sorted) CMTRX, where the main diagonal guides the interpretation process (Congalton & Green, 1999), to make it more intuitive and familiar to RS practitioners. In general, no binary relationship R: A ⇒ B between an *a priori* vocabulary A of static sub-symbolic color names and a user- and application-dependent dictionary B of symbolic classes of observed objects in the scene-domain is a bijective function, refer to Table 3 and Equation (3). As a consequence of its unrealistic hypothesis in color information/knowledge representation, the 1D image classification approach proposed in Salmon et al. (2013) scored low in accuracy. Unfortunately, to explain their poor MS image classification outcome these authors concluded that, in their experiments, a year 2006 SIAM’s static dictionary of color names was useless to identify target LC classes. The lesson to be gained by these authors’ experience is that well-established RS practices, such as 1D image analysis based on supervised data learning algorithms and thematic map quality assessment by means of a square and sorted CMTRX where test and reference thematic legends are the same, can become malpractices when an *a priori* dictionary of static color names is employed for MS image classification purposes in agreement with Equation (3) and common sense, see Table 3. This lesson learned is supported by the fact that one of the same co-authors of paper (Salmon et al., 2013) reached opposite conclusions when a year 2013 SIAM application software, the same investigated by the present paper, was employed successfully in detecting burned areas from MS image time-series according to a convergence of color names with spatio-temporal visual properties in agreement with Equation (3) (Boschetti et al., 2015).

## 4. Original hybrid eight-step guideline for identification of a categorical variable-pair binary relationship

Summarized in Chapter 1, our experimental project required to compare an annual time-series of test SIAM-WELD maps of sub-symbolic color names, see Figure 6, with a reference USGS NLCD 2006 map whose legend of symbolic LC classes is summarized in Table 2. Since these test and reference map legends do not coincide, they must be reconciled/harmonized through a binary relationship R: VocabularyOfColorNames ⇒ LegendOfObjectClassNames (and vice versa), refer to Equation (3).

The harmonization of ontologies and the comparison of thematic maps with different legends are the subject of research of a minor body of literature, e.g., refer to works in ontology-driven geographic information systems (ODGIS) (Fonseca, Egenhofer, Agouris, & Camara, 2002; Guarino, 1995; Sowa, 2000). Ahlqvist writes that “to negotiate and compare information stemming from different classification systems (Bishr, 1998; Mizen, Dolbear, & Hart, 2005)… a translation can be achieved by *matching the concepts in one system with concepts in another*, either directly or through an intermediate classification (Feng & Flewelling, 2004; Kavouras & Kokla, 2002)” (Ahlqvist, 2005). Stehman describes four common types of thematic map-pair comparisons (Stehman, 1999). In the first type, different thematic maps, either crisp or fuzzy, of the same region of interest and employing the same sorted set (legend) of LC classes are compared (Kuzera & Pontius, 2008). In the second type, which includes the first type as a special case, thematic maps, either crisp of fuzzy, of the same region of interest, but featuring map legends that differ in their basic terms with regard to semantics and/or cardinality and/or order of presentation are compared. The third and fourth types of thematic maps comparison regard maps of different surface areas featuring, respectively, the same dictionary or different dictionaries of basic terms. Whereas a large portion of the RS community appears concerned with the aforementioned first type of map comparisons exclusively, the protocol proposed in (Baraldi et al., 2014) focuses on the second type, which includes the first type as a special case. In Couclelis (2010), the author observed that inter-dictionary concept matching (“conceptual matching”) (Ahlqvist, 2005) is an inherently equivocal *information-as-data-interpretation* process (Capurro & Hjørland, 2003), see Table 3. In common practice, two independent human domain-experts (cognitive agents, knowledge engineers) are likely to identify different binary associations between two codebooks of codewords (Laurini & Thompson, 1992). The conclusion is that no “universal best match” of two different codebooks can exist, but identification of the most appropriate binary relationship between two different nomenclatures becomes a subjective matter of negotiation to become community-agreed upon (Baraldi, 2017; Capurro & Hjørland, 2003; Couclelis, 2010).

To streamline the inherently subjective selection of “correct” entry-pairs in a binary relationship R: A ⇒ B ⊆ A × B between two univariate categorical variables A and B estimated from a single population, an original hybrid eight-step guideline was designed for best practice, where deductive/top-down prior beliefs and inductive/bottom-up learning-from-data inference are combined. This hybrid protocol is sketched hereafter as the second original and pragmatic contribution of the present Part 1 to fill the gap from EO sensory data, mapped onto BC names, to ESA EO Level 2 product, whose SCM features semantics. As an example, let us consider a binary relationship R: A ⇒ B = VocabularyOfColorNames ⇒ LegendOfObjectClassNames ⊆ A × B where rows are a test set of three semi-symbolic color names, say, A = {MS green-as-“*Vegetation*”, MS white-as-“*Cloud*”, “*Unknowns*”}, where |A| = a = ColorVocabularyCardinality* = TC* = 3 is the row (test) cardinality, and where columns are a reference set of three symbolic LC classes, say, B = { “*Evergreen Forest*”, “*Deciduous Forest*”, “*Others*”}, where |B| = b = ObjectClassLegendCardinality = *RC* = 3 is the column (reference) cardinality.

1. Display multivariate frequency distributions of the two univariate categorical variables estimated from a single population in the BIVRFTAB = FrequencyCount(A × B) whose size is *TC* × *RC*.
2. Estimate probabilities in the BIVRFTAB cells.
3. Compute class-conditional probability *p*(*r* | *t*) of reference class *r* = *1*, …, *RC*, given test class *t* = 1, …, *TC*.
4. Reset to zero all *p*(*r* | *t*) below *TH1*∈ [0, 1] (e.g., *TH1* = 9%), otherwise set that cell to 1. Let us identify this contingency table instantiation as *DataDrivenConditionalProb*(*r*|*t*)(x, y), x = *1*, …, *RC*, y = 1, …, *TC*.
5. Compute class-conditional probability *p*(*t* | *r*) of test class *t* = 1, …, *TC*, given reference class *r* = 1, …, *RC*.
6. Reset to zero all *p*(*t* | *r*) below *TH2* ∈ [0, 1] (e.g., *TH2 *= 6% ≤ *TH1*), otherwise set that cell to 1. Let us identify this contingency table instantiation as *DataDrivenConditionalProb*(*t*|*r*)(x, y), x = *1*, …, *RC*, y = 1, …, *TC*.
7. Compute *DataDrivenTemporaryCells*(x, y) = max{*DataDrivenConditionalProb*(*t*|*r*)(x, y), *DataDrivenConditionalProb*(*r*|*t*)(x, y)}, x = *1*, …, *RC*, y = 1, …, *TC*. At this point, based exclusively on bottom-up evidence stemming from frequency data, in the 2-fold Cartesian product A × B each cell is equal to 0 or 1. Then that cell is termed either “temporary non-correct” or “temporary correct”.
8. Top-down scrutiny by a human domain-expert of each cell in the BIVRFTAB, which is either “temporary correct” or “temporary non-correct” at this point, to select those cells to be finally considered as “correct entry-pairs”. Actions undertook by this top-down scrutiny are twofold.
  - Switch any data-derived “temporary correct” cell to a “final non-correct” cell if it is provided with a strong prior belief of conceptual mismatch. For example, based on experimental evidence a test spectral category MS white-as-“*Cloud*” can match a reference LC class “*Evergreen Forest*”: this data-derived entry-pair match must be considered non-correct in the final R: A ⇒ B following semantic scrutiny by a human expert.
  - Switch any data-derived “temporary non-correct” cell to a “final correct” cell if it is provided with a strong prior belief of conceptual match. For example, the test spectral category MS green-as-“*Vegetation*” is considered a superset of the reference LC class “*Deciduous Forest*” irrespective of whether there are frequency data in support of this conceptual relationship.

Table 5 shows an example of how this protocol can be employed in practice. In Table 5 the last step 8 identifies an inherently equivocal *information-as-data-interpretation* process, where a human decision maker has a pro-active role in providing frequency data with semantics (symbolic meanings) (Capurro & Hjørland, 2003). It is highly recommended that any inherently subjective *information-as-data-interpretation* activity occurs as late as possible in the information processing workflow, to avoid propagation of “errors” due to personal preferences not yet community-agreed upon. Noteworthy, in the proposed eight-step guideline there are two “hidden” system’s free-parameters to be user-defined based on heuristics, equivalent to a trial-and-error strategy: variables *TH1* and *TH2* are two numeric thresholds in range [0, 1] for binary (hard, crisp) decision making, whose normalized range of change and intuitive meaning in terms of probability should make their selection easy and, to a certain extent, application- and user-independent.

## 5. Original measure of association (harmonization) in a categorical variable-pair binary relationship eligible for guiding the interpretation process of a two-way contingency table

Traditional scalar indicators of bivariate categorical variable association estimated from a BIVRFTAB = FrequencyCount(A × B), either square or non-square, include the Pearson’s chi-square index of statistical independence and the normalized Pearson’s chi-square index, also known as Cramer’s coefficient V (Sheskin, 2000). These frequentist statistics of independence do not apply to a binary relationship R: A ⇒ B ⊆ A × B, such as that shown in Table 3, where there is no frequency count, i.e., binary relationship R: A ⇒ B ⊆ A × B ≠ FrequencyCount(A × B) = BIVRFTAB, refer to Chapter 4. Hereafter, a scalar indicator of association (harmonization, reconciliation) between two univariate categorical variables (codebooks of codewords), A and B, collected from a single population, called Categorical Variable Pair Association Index (CVPAI) in range [0, 1], is estimated from a binary relationship R: A ⇒ B, such that CVPAI(R: A ⇒ B) ∈ [0, 1].

**Figure 18.**
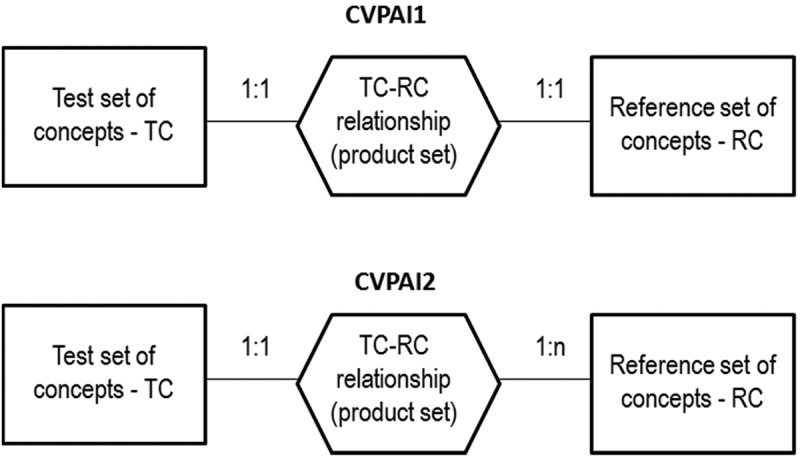
Entity-relationship conceptual model representation of the binary relationship R: A ⇒ B from set A = test categorical variable to set B = reference categorical variable, provided with the min:max cardinality required by the Categorical Variable-Pair Association Index (CVPAI) formulation 1 (CVPAI1) and formulation 2 (CVPAI2) to score maximum in range [0, 1]. Inequality CVPAI1 ≤ CVPAI2 holds, i.e., the latter is a relaxed version of the former. In particular, CVPAI1 is maximum (equal 1) when the binary relationship R: A ⇒ B from set A = test categorical variable to set B = reference categorical variable is a bijective function, both injective (one-to-one) and surjective (onto). CVPAI2 is maximum when the binary relationship R: A ⇒ B is either a surjective function or a bijective function.

Proposed in Baraldi et al. (2014), a CVPAI version 1, CVPAI1(R: A ⇒ B) ∈ [0, 1], is maximized (tends to 1, meaning maximum harmonization) if the binary relationship R: A ⇒ B from set A = test categorical variable, e.g., vocabulary of color names, to set B = reference categorical variable, e.g., dictionary of LC class names, is a bijective function, i.e., the binary relationship R: A ⇒ B is a function, therefore to each instance in test set A (of color names) corresponds a single instance in reference set B (of LC class names), and this function is both injective (one-to-one, for any instance in reference set B of LC class names there is no more than one instance in test set A of color names) and surjective (onto, for any instance in reference set B of LC class names there is at least one instance in test set A of color names), see Figure 18.

Hereafter, original formulations of CVPAI version 2, CVPAI2(R: A ⇒ B) ∈ [0, 1], and CVPAI version 3, CVPAI3(R: A ⇒ B) ∈ [0, 1], complementary not alternative to the CVPAI1 formulation presented in (Baraldi et al., 2014), are proposed as the third original and analytic contribution of the present Part 1 of this paper. Unlike the CVPAI1 expression, a novel CVPAI2 formulation was constrained as follows, see Figure 18. (i) The “most discriminative” test-to-reference inter-set binary relation R: A ⇒ B is a function, i.e., each test color name in test set A matches with only one reference LC class name in reference set B. (ii) The “most discriminative” reference-to-test class relation is either a surjective function, i.e., each reference LC class in set B matches with at least one test color name in set B, or a bijective function, both surjective and injective as a special case of the former, i.e., each reference LC class in set B matches with only one test color name in set A, see Figure 18. In short, CVPAI2(R: A ⇒ B) is maximum (tends to 1, meaning maximum harmonization) when the binary relationship R: A ⇒ B is either a surjective function or a bijective function. Since the CVPAI2 formulation relaxes the CVPAI1 formulation, see Figure 18, it is always true that CVPAI2(R: A ⇒ B) ≥ CVPAI1(R: A ⇒ B) ∈ [0, 1]. The two formulations CVPAI1 and CVPAI2 are complementary not alternative because they have been designed to be maximized by different distributions of “correct” entry-pair cells in a binary relationship R: A ⇒ B ⊆ A × B, see Figure 18. Both formulations CVPAI1(R: A ⇒ B) and CVPAI2(R: A ⇒ B) are independent of frequency counts generated by a bivariate categorical variable distribution to be displayed in a BIVRFTAB = FrequencyCount(A × B) ≠ R: A ⇒ B ⊆ A × B. Depending on the problem at hand, where two specific instantiations of test set A and reference set B are estimated from the same population, either CVPAI1 or CVPAI2 can be considered better suited for estimating the degree of harmonization between sets A and B, see Figure 18. If test set A is a vocabulary of BC names and reference test B is a legend of LC class names, then the novel CVPAI2 formulation is recommended for use. An alternative formulation to CVPAI2(R: A ⇒ B) is CVPAI3(R: A ⇒ B). They are both maximized by the same distribution of “correct” entry-pair cells in a binary relationship R: A ⇒ B ⊆ A × B.

The analytic formulation of CVPAI2(R: A ⇒ B), see Figure 18, is proposed as follows. In a binary relationship R: A ⇒ B ⊆ A × B, set A is a test codebook of cardinality |A| = *TC* as rows and set B is a reference codebook of cardinality |B| = *RC* as columns, so that the size of the 2-fold Cartesian product A × B is *TC*×*RC*. The total number of ”correct” entry-pair cells in R: A ⇒ B is identified as *CE*, where 0 ≤ *CE* ≤ *TC*×*RC*. In addition, symbol “ = = ” is adopted to mean “equal to”. The CVPAI2 formulation is constrained as follows.

-
  $$\;\;\;\begin{array}{c} CE=\overset{TC}{\underset{t=1}{\sum }}\overset{RC}{\underset{r=1}{\sum }}CE_{t,r},with\;CE_{t,r}\in \left\{0,1\right\}=\\ \left\{non-correct\;entry-pair(t,r)=0,\;correct\;entry-pair(t,r)=1\right\},\\ CE\in \left\{0,RC\times TC\right\}. \end{array}$$
- If (*CE* = = 0) then *CVPAI2* = 0 must hold. It means that, when no “correct’” cell exists, then the degree of conceptual match between the two categorical variables is zero.
- If (*CE* = = *TC*×*RC*) then *CVPAI2* → 0 must hold. It means that when all table cells are considered “correct”, then no entry-pair is discriminative (informative), i.e., nothing makes the difference between the two categorical variables.
- If

$$\;\;\left\{\begin{array}{c} \left[\left(\overset{TC}{\underset{t=1}{\sum }}CE_{t,r}=CE_{+,r}\right)>0,\;r=1,\ldots ,RC\right]AND\\ \left[\left(\overset{RC}{\underset{r=1}{\sum }}CE_{t,r}=CE_{t,+}\right)==1,t=1,\ldots ,TC\right] \end{array}\right\},$$

where *CE_+,r_* is the total sum of correct entry-cell pairs along reference column *r*, with *r* = 1, …, *RC, CE_t,+_* is the total sum of correct entry-cell pairs along test row *t*, with *t* = 1, …, *TC*, then *CVPAI2* must be maximum, i.e., *CVPAI2* = 1. It means that if for each test class *t* = 1, …, *TC* there is one single match and for each reference class *r* = 1, …, *RC* there is at least one match, then *CVPAI2* must be maximum, such that *CVPAI2* = 1, see Figure 18.

(e) If [*not* condition(b) *AND not* condition(c) *AND not* condition(d)] then *CVPAI2* ∈ (0,1).

In a square binary relationship R: A ⇒ B where *TC* = = *RC*, to maximize the CVPAI2 (to become equal to 1), submitted to condition (d), the binary relationship must be a 1–1 function (an injective function, 1–1 forward and 1–1 backward). To satisfy the set of aforementioned constraints (a) to (e), the following set of original equations is proposed.

(5) $$CVPAI2\;\in \left[0,1\right],\;CVPAI2=\frac{1}{RC+TC}\left(\overset{RC}{\underset{r=1}{\sum }}f_{RC}(CE_{+,r})+\overset{TC}{\underset{t=1}{\sum }}f_{TC}(CE_{t,+})\right),\;$$

with

(6) $$f_{RC}(i)=\left\{ \begin{array}{l} 0ifi=0,\\ 1ifi>0,\; \end{array}\right.i\in \left\{0,TC\right\}\subset {\text{I}}_{0}^{+},\text{where}i=CE_{+,r},r\in \left\{1,RC\right\},$$

(7) $$f_{TC}(j)=\left\{ \begin{array}{l} 0ifj=0,\\ GaussianMembership(j,Center=1,StnDev=RC/3)=\\ e^{-\;\frac{1}{2}\frac{{\left(j-1\right)}^{2}}{{\left(\frac{RC}{3}\right)}^{2}}}\in [0,1],\;ifj>0,\text{with}j\in \left\{0,RC\right\}\subset {\text{I}}_{0}^{+},\text{where}j=CE_{t,+},t\in \left\{1,TC\right\}. \end{array}\right.$$

Although it is maximized by the same distribution of “correct” entry-pair cells in a binary relationship R: A ⇒ B ⊆ A × B, a novel CVPAI3 expression is a more severe formulation of CVPAI2, i.e., 1 ≥ CVPAI2 ≥ CVPAI3 ∈ [0, 1]. The proposed CVPAI3 formulation alternative to CVPAI2 is the following.

(8) $$CVPAI3\;\in \left[0,1\right],\;CVPAI3=\min\left\{\frac{\overset{RC}{\underset{r=1}{\sum }}f_{RC}(CE_{+,r})}{RC},\frac{\overset{TC}{\underset{t=1}{\sum }}f_{TC}(CE_{t,+})}{TC}\right\}.\;$$

In Equation (7), GaussianMembership(j, Center = 1, StnDev = RC/3) → 0 when it covers approximately 99.73% of the area underneath the Gaussian curve at distance j ≈ ± 3 ∙ StnDev = ± 3 ∙ RC/3 = ± RC from its Center = 1. If j = 1, then GaussianMembership(j, Center = 1, StnDev = RC/3) = 1. It is trivial to prove that Equations (5–7) satisfy the aforementioned requirements (a) to (d). By means of a numeric example, it can be shown that requirement (e) is satisfied too. For example, estimated from the binary relationship instantiated at step 8 of Table 5, CVPAI2 = (1/6)*(1 + 1 + 1 + 1 + 1 + exp(−0.5*(3–1)^2/(3/3)^2)) = (1/6) * (5 + 0.1353) = 0.8558. Intuitively closer to 1 than 0, this CVPAI2 value shows that the harmonization between the two test and reference nominal variables is (fuzzy) “high” (>0.8) in Table 5.

To appreciate the conceptual difference between the CVPAI1 and CVPAI2 formulations maximized by different distribution of “correct” entry-pairs in a binary relationship R: A ⇒ B ⊆ A × B, see Figure 18, let us compare a test vocabulary A of color names, such as SIAM’s, see Figure 6, with a reference dictionary B of LC class names, such as the USGS NLCD’s, see Table 2. In terms of capability of color names to discriminate LC class names, the ideal test-to-reference binary relationship is a function where one color name matches with only one reference LC class. On the other hand, the color attribute of a real-world LC class can be typically linked to one or more discrete color names, see Table 3. In this realistic example the range of change of an estimated CVPAI2 value would be (0, 1], up to its maximum value equal to 1, while the range of change of the CVPAI1 formulation proposed in (Baraldi et al., 2014) would be (0, 1), below its maximum value equal to 1, see Figure 18.

Another example where the difference between the CVPAI1 and CVPAI2 formulations is highlighted is when the test dictionary A is a specialized version of the reference dictionary B, according to a parent–child relationship. For example, a test taxonomy of LC classes is A = LegendOfObjectClassNames_A = {LC class “*Dark-tone bare soil”*, LC class “*Light-tone bare soil”*, LC class “*Deciduous Forest”*, LC class “*Evergreen Forest”*} and a reference LC class taxonomy is B = LegendOfObjectClassNames_B = {LC class “*Bare soil”*, LC class “*Forest”*}. Based on our prior knowledge-based understanding of these two semantic dictionaries A and B, a reasonable binary relationship can be considered R: A ⇒ B = {(LC class “*Dark-tone bare soil*”, LC class “*Bare soil*”); (LC class “*Light-tone bare soil*”, LC class “*Bare soil*”); (LC class “*Deciduous Forest*”, LC class “*Forest*”); (LC class “*Evergreen Forest*”, LC class “*Forest*”)}. In this case, the CVPAI1 formulation scores below its maximum, i.e., CVPAI1 ∈ (0, 1), while the expected CVPAI2 value would score maximum, i.e., CVPAI2 = 1, meaning that the two vocabularies are harmonized because one is the specialization of the other, featuring a parent–child relationship.

These two examples illustrate the intuitive meaning and practical use of the normalized quantitative indicator CVPAI2 ∈ [0, 1] in an EO-IUS implementation based on a convergence-of-evidence approach, in agreement with Equation (3). When the semantic information gap from sub-symbolic sensory data to a symbolic set B = LegendOfObjectClassNames is filled by an EO-IUS starting from a static color naming first stage, provided with a semi-symbolic set A = VocabularyOfColorNames, if the binary relationship R: A ⇒ B ⊆ A × B features a degree of association CVPAI2 ∈ [0, 1], then (1—CVPAI2) ∈ [0, 1] is the semantic information gap from sub-symbolic sensory data to the symbolic LegendOfObjectClassNames left to be filled by further stages in the hierarchical EO-IUS pipeline, where spatial information is masked by first-stage color names. If CVPAI2 = 1, then secondary color information discretized by set A = VocabularyOfColorNames suffices to detect target set B = LegendOfObjectClassNames with no further need to investigate primary spatial information in a hierarchical convergence-of-evidence image classification approach, refer to Equation (3).

## 6. Conclusions

To pursue the GEO-CEOS visionary goal of a GEOSS implementation plan for years 2005–2015 not-yet accomplished by the RS community, this interdisciplinary work aimed at filling an analytic and pragmatic information gap from EO image big data to systematic ESA EO Level 2 product generation at the ground segment, never achieved to date by any EO data provider and postulated as necessary not sufficient pre-condition to GEOSS development. For the sake of readability this paper is split into two, the present Part 1 - Theory and the following Part 2 - Validation.

The original contribution of the present Part 1 is fourfold. A first lesson was learned from published works on prior knowledge-based MS reflectance space hyperpolyhedralization into static (non-adaptive-to-data) color names, according to the principle of color naming discovered by linguistics and investigated by CV in the realm of cognitive science, see Figure 13. In color naming, a static vocabulary of sub-symbolic color names is equivalent to a set of mutually exclusive and totally exhaustive (hyper)polyhedra, neither necessarily convex nor connected, in a color data (hyper)cube. It was observed that well-established RS practices, such as 1D image analysis based on supervised data learning algorithms, where dominant spatial information is neglected in favor of secondary color information, and thematic map quality assessment where test and reference map legends are required to coincide, can become malpractices when an *a priori* dictionary of static color names is employed for MS image classification based on a convergence-of-evidence approach, such as in Bayesian naïve classification, see Equation (3). When test and reference thematic map legends A and B are the same, the binary relationship R: A ⇒ B ⊆ A × B becomes a bijective function (both 1–1 and onto) and the main diagonal of the 2-fold Cartesian product A × B guides the interpretation process of a bivariate frequency table, BIVRFTAB = FrequencyCount(A × B), equal to a square and sorted confusion matrix, CMTRX. This constraint makes a CMTRX, whose input categorical variables A and B coincide, intuitive to understand and more familiar to RS practitioners. Noteworthy, inequality R: A ⇒ B ⊆ A × B ≠ FrequencyCount(A × B) = BIVRFTAB always holds true, where one instance of the binary relationship R guides the interpretation process of the two-way contingency table BIVRFTB. Quite surprisingly, the non-coincident assumption between an *a priori* vocabulary A of static sub-symbolic color names in the measurement color space and a user- and application-dependent legend B of symbolic classes of real-world objects in the scene-domain, where inequality A ≠ B always holds, appears somehow difficult to acknowledge by relevant portions of the RS community, in contrast with common sense, see Table 3.

Second, Equation (3) was proposed as an analytic expression of a biologically plausible hybrid (combine deductive and inductive) CV system suitable for convergence of color and spatial evidence, in agreement with a Bayesian approach to vision proposed by Marr (1982), with the principle of statistic stratification well known in statistics and with the divide-and-conquer (*divide-et-impera*) problem solving criterion widely adopted in structured engineering. In compliance with common sense, see Table 3, Equation (3) shows that a static color naming first stage can be employed for stratification purposes of further spatial-context sensitive image classification stages. In the static color naming first stage, a binary relationship R: A ⇒ B ⊆ A × B from a vocabulary A of general-purpose static color names in the MS color space to a taxonomy B of LC class names in the 4D spatio-temporal scene-domain, such as the standard FAO LCCS taxonomy shown in Figure 3, can be established by human experts based on top-down prior beliefs, if any, in combination with bottom-up evidence inferred from new data, as described in Table 5. Once established and community-agreed upon, a binary relationship R: A ⇒ B ⊆ A × B from a vocabulary A of static color names in a MS color space to a standard legend of LC classes in the 4D spatio-temporal scene-domain becomes equivalent to an *a priori* knowledge base in a Bayesian updating framework, where Bayesian inference is applied iteratively: after observing some evidence, the resulting posterior probability can be treated as prior probability. For example, once community-agreed upon, Table 3 becomes equivalent to an *a priori* knowledge base available in addition to sensory data, and a new posterior probability can be computed from new data, e.g., to pursue image classification in agreement with Equation (3).

Third, for best practice a hybrid eight-step protocol, sketched in Table 5, was proposed to infer a binary relationship, R: A ⇒ B ⊆ A × B, from categorical variable A to categorical variable B estimated from the same population, where codebooks A and B can differ in cardinality, semantics or in the order of presentation of codewords. This protocol streamlines a hybrid combination of deductive prior beliefs by human domain experts with inductive evidence from data. It is of practical use because identification of a binary relationship R: A ⇒ B is mandatory to guide the interpretation process of a bivariate frequency table, BIVRFTAB = FrequencyCount(A × B), where A ≠ B in general. Only if A = B then BIVRFTAB becomes equal to the well-known square and sorted CMTRX, where the main diagonal guides the interpretation process.

Fourth, in compliance with the GEO-CEOS QA4EO *Val* guidelines, two original and alternative formulations, CVPAI2(R: A ⇒ B ⊆ A × B) ∈ [0, 1] and CVPAI3(R: A ⇒ B ⊆ A × B) ∈ [0, 1], were proposed as categorical variable-pair degree of association (harmonization) in a binary relationship, R: A ⇒ B, from categorical variable A to categorical variable B estimated from the same population, where A ≠ B in general. When CVPAI2 or CVPAI3 is maximum, equal to 1, then the two categorical variables A and B are considered fully harmonized.

To comply with the GEO-CEOS QA4EO *Cal*/*Val* requirements, the subsequent Part 2 of this paper presents and discusses a GEO-CEOS stage 4 *Val* of the annual SIAM-WELD map time-series for the years 2006 to 2009 in comparison with the reference USGS NLCD 2006 map, based on an original protocol for wall-to-wall inter-map comparison without sampling where the test and reference maps feature the same spatial resolution and spatial extent, but whose legends are not the same and must be harmonized.

## Abbreviations

AI: Artificial Intelligence
ATCOR: Atmospheric/Topographic Correction commercial softare product
AVHRR: Advanced Very High Resolution Radiometer
BC: Basic Color
BIVRFTAB: Bivariate Frequency Table
Cal: Calibration
Cal/Val: Calibration and Validation
CCRS: Canada Centre for Remote Sensing
CEOS: Committee on Earth Observation Satellites
CLC: CORINE Land Cover (taxonomy)
CMTRX: Confusion Matrix
CNES: Centre national d’études spatiales
CONUS: Conterminous U.S.
CORINE: Coordination of Information on the Environment
CV: Computer Vision
CVPAI: Categorical Variable Pair Association Index (in range [0, 1])
DCNN: Deep Convolutional Neural Network
DLR: Deutsches Zentrum für Luft- und Raumfahrt (German Aerospace Center)
DN: Digital Number
DP: Dichotomous Phase (in the FAO LCCS taxonomy)
DRIP: Data-Rich, Information-Poor (scenario)
EDC: EROS Data Center
EO-IU: EO Image Understanding
EO-IUS: EO-IU System
EPA: Environmental Protection Agency
EROS: Earth Resources Observation Systems
ESA: European Space Agency
FAO: Food and Agriculture Organization
GEO: Intergovernmental Group on Earth Observations
GEOSS: Global EO System of Systems
GIGO: Garbage In, Garbage Out principle of error propagation
GIS: Geographic Information System
GIScience: Geographic Information Science
GUI: Graphic User Interface
HS: Hyper-Spectral
IGBP: International Global Biosphere Programme
IUS: Image Understanding System
LC: Land Cover
LCC: Land Cover Change
LCCS: Land Cover Classification System (taxonomy)
LCLU: Land Cover Land Use
LEDAPS: Landsat Ecosystem Disturbance Adaptive Processing System
mDMI: Minimally Dependent and Maximally Informative (set of quality indicators)
MHP: Modular Hierarchical Phase (in the FAO LCCS taxonomy)
MIR: Medium InfraRed
MODIS: Moderate Resolution Imaging Spectroradiometer
MS: Multi-Spectral
NASA: National Aeronautics and Space Administration
NIR: Near InfraRed
NLCD: National Land Cover Data
NOAA: National Oceanic and Atmospheric Administration
OA: Overall Accuracy
OAMTRX: Overlapping Area Matrix
OBIA: Object-Based Image Analysis
ODGIS: Ontology-Driven GIS
OGC: Open Geospatial Consortium
OP: Outcome (product) and Process
OP-Q^2^I: Outcome and Process Quantitative Quality Index
QA: Quality Assurance
QA4EO: Quality Accuracy Framework for Earth Observation
Q^2^I: Quantitative Quality Indicator
R&D: Research and Development
RGB: monitor-typical Red-Green-Blue data cube
RMSE: Root Mean Square Error
RS: Remote Sensing
SCM: Scene Classification Map
SEN2COR: Sentinel 2 (atmospheric) Correction Prototype Processor
SIAM™: Satellite Image Automatic Mapper™
STRATCOR: Stratified Topographic Correction
SS: Super-Spectral
SURF: Surface Reflectance
TIR: Thermal InfraRed
TOA: Top-Of-Atmosphere
TOARF: TOA Reflectance
TM (superscript): (non-registered) Trademark
UML: Unified Modeling Language
USGS: US Geological Survey
Val: Validation
VQ: Vector Quantization
WELD: Web-Enabled Landsat Data set
WGCV: Working Group on Calibration and Validation

## Figures and Tables

**Figure:**
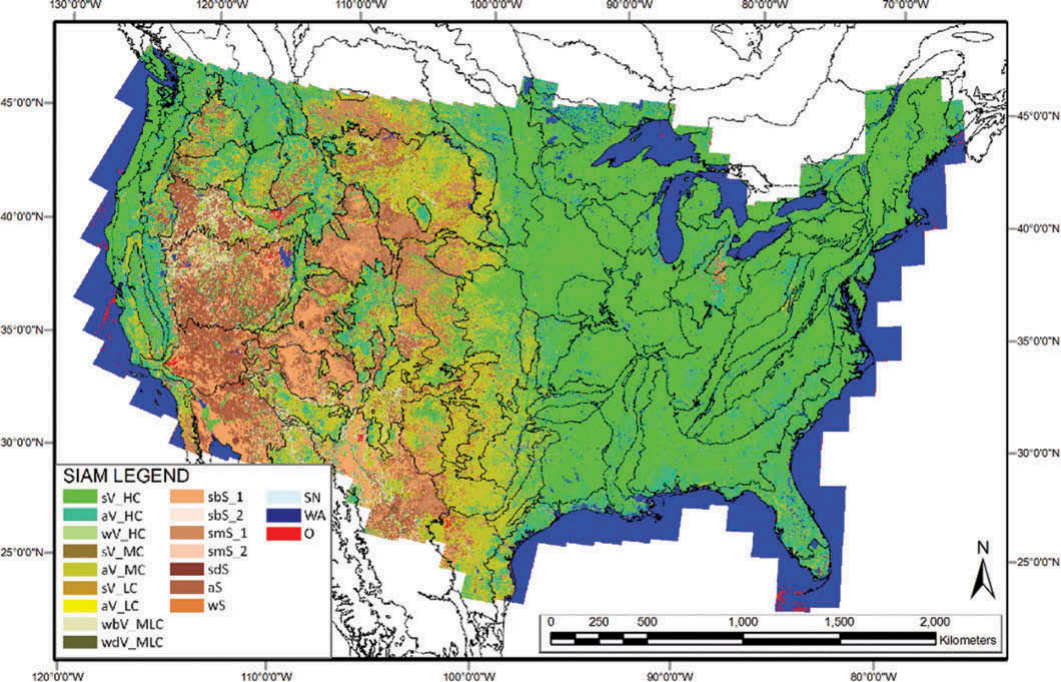


## Cover image

Source: Satellite Image Automatic Mapper (SIAM) multi-level map generated automatically, without human-machine interaction, and in near real-time, specifically, in linear time complexity with image size, from the 30 m resolution annual Web-Enabled Landsat Data (WELD) image composite for the year 2006 of the conterminous U.S. (CONUS), radiometrically calibrated into top-of-atmosphere reflectance (TOARF) values. The multi-level map’s legend, shown at bottom left, is the SIAM intermediate discretization level, consisting of 48 basic color (BC) names reassembled into 19 spectral macro-categories by an independent human expert, according to a proposed hybrid (combined deductive and inductive) eight-step protocol for identification of a categorical variable-pair binary relationship, from a vocabulary of BC names to a dictionary of land cover (LC) class names. No discrete and finite vocabulary of color names, such as SIAM’s, equivalent to a set of mutually exclusive and totally exhaustive (hyper)polyhedra, neither necessarily convex nor connected, in a MS reflectance (hyper)space, should ever be confused with a symbolic (semantic) taxonomy of LC class names in the 4D geospace-time scene-domain. Black lines across the SIAM-WELD 2006 color map represent the boundaries of the 86 Environmental Protection Agency (EPA) Level III ecoregions of the CONUS, suitable for regional-scale statistical stratification required to intercept geospatial non-stationary statistics, typically lost when a global spatial average, e.g., at continental spatial extent, is superimposed on the local computational processes.
